# Patient Priorities–Aligned Care for Older Adults With Multiple Conditions: A Nonrandomized Controlled Trial

**DOI:** 10.1001/jamanetworkopen.2023.52666

**Published:** 2024-01-23

**Authors:** Mary E. Tinetti, Ardeshir Hashmi, Henry Ng, Margaret Doyle, Toyomi Goto, Jessica Esterson, Aanand D. Naik, Lilian Dindo, Fan Li

**Affiliations:** 1 Department of Medicine, Yale School of Medicine, New Haven, Connecticut; 2 Department of Chronic Disease Epidemiology, Yale School of Public Health, New Haven, Connecticut; 3 Center for Geriatric Medicine, Cleveland Clinic, Cleveland, Ohio; 4 Department of Internal Medicine, Cleveland Clinic, Cleveland, Ohio; 5 Center for Value-Based Care Research, Cleveland Clinic, Cleveland, Ohio; 6 Institute on Aging, University of Texas Health Science Center, Houston; 7 Houston VA HSR&D Center for Innovations in Quality, Effectiveness and Safety, Michael E DeBakey Veterans Affairs Medical Center, Houston, Texas; 8 Department of Medicine, Health Services Research, Baylor College of Medicine, Houston, Texas; 9 Department of Biostatistics, Yale School of Public Health, New Haven, Connecticut; 10 Center for Methods in Implementation and Prevention Science, Yale School of Public Health, New Haven, Connecticut

## Abstract

**Importance:**

Older adults with multiple conditions receive health care that may be burdensome, of uncertain benefit, and not focused on what matters to them. Identifying and aligning care with patients’ health priorities may improve outcomes.

**Objective:**

To assess the association of receiving patient priorities care (PPC) vs usual care (UC) with relevant clinical outcomes.

**Design, Setting, and Participants:**

In this nonrandomized controlled trial with propensity adjustment, enrollment occurred between August 21, 2020, and May 14, 2021, with follow-up continuing through February 26, 2022. Patients who were aged 65 years or older and with 3 or more chronic conditions were enrolled at 1 PPC and 1 UC site within the Cleveland Clinic primary care multisite practice. Data analysis was performed from March 2022 to August 2023.

**Intervention:**

Health professionals at the PPC site guided patients through identification of values, health outcome goals, health care preferences, and top priority (ie, health problem they most wanted to focus on because it impeded their health outcome goal). Primary clinicians followed PPC decisional strategies (eg, use patients’ health priorities as focus of communication and decision-making) to decide with patients what care to stop, start, or continue.

**Main Outcomes and Measures:**

Main outcomes included perceived treatment burden, Patient-Reported Outcomes Measurement Information System (PROMIS) social roles and activities, CollaboRATE survey scores, the number of nonhealthy days (based on healthy days at home), and shared prescribing decision quality measures. Follow-up was at 9 months for patient-reported outcomes and 365 days for nonhealthy days.

**Results:**

A total of 264 individuals participated, 129 in the PPC group (mean [SD] age, 75.3 [6.1] years; 66 women [48.9%]) and 135 in the UC group (mean [SD] age, 75.6 [6.5] years; 55 women [42.6%]). Characteristics between sites were balanced after propensity score weighting. At follow-up, there was no statistically significant difference in perceived treatment burden score between groups in multivariate models (difference, −5.2 points; 95% CI, −10.9 to −0.50 points; *P* = .07). PPC participants were almost 2.5 times more likely than UC participants to endorse shared prescribing decision-making (adjusted odds ratio, 2.40; 95% CI, 0.90 to 6.40; *P* = .07), and participants in the PPC group experienced 4.6 fewer nonhealthy days (95% CI, −12.9 to −3.6 days; *P* = .27) compared with the UC participants. These differences were not statistically significant. CollaboRATE and PROMIS Social Roles and Activities scores were similar in the 2 groups at follow-up.

**Conclusions and Relevance:**

This nonrandomized trial of priorities-aligned care showed no benefit for social roles or CollaboRATE. While the findings for perceived treatment burden and shared prescribing decision-making were not statistically significant, point estimates for the findings suggested that PPC may hold promise for improving these outcomes. Randomized trials with larger samples are needed to determine the effectiveness of priorities-aligned care.

**Trial Registration:**

ClinicalTrials.gov Identifier: NCT04510948

## Key Points

**Question:**

Is the receipt of health priorities–aligned primary care associated with patient-reported and health care utilization outcomes for older adults with multiple chronic conditions?

**Findings:**

In this nonrandomized controlled trial of 264 individuals, participants receiving patient priorities care vs usual care did not have statistically significantly different treatment burden scores, odds of shared medication decision-making, and number of nonhealthy days. There was no difference in perception of whether their care was goal directed.

**Meaning:**

While these findings suggest that aligning care with health priorities may help improve some important health outcomes for older adults, further research is needed to determine potential effects of priorities-aligned care.

## Teaser

This nonrandomized controlled trial compares health care aligned with patient values, goals, and priorities with usual care to improve clinical outcomes for older adults with multiple conditions.

## Introduction

Older adults with multiple chronic conditions (MCCs) are major users of health care.^1^ This health care may be burdensome, of uncertain benefit and potential harm, and not aligned with individuals’ health priorities.^2,3,4,5,6,7,8^ Persons with MCCs are known to vary in their most desired health outcomes and in the health care they are willing and able to receive to achieve these outcomes.^6,7,8,9,10^

We developed patient priorities care (PPC) with input from patients, care partners, clinicians, and health system leaders and payers to address these issues.^11,12,13,14,15,16^ PPC focuses care on achieving patients’ health goals within the context of their health conditions and the health care they are willing and able to receive. The underlying premise is to move siloed disease-based decision-making from “You need (intervention) to (prevent, treat, manage) (disease)” to “Knowing your health conditions, overall health, and what matters most to you, I suggest we (intervention) and see if it helps (patient goal).”

Involving both patients and clinicians, PPC begins with a structured process in which patients work with a member of the health care team to identify the health outcome goals they most desire to achieve and to specify what they are willing (or unwilling) to do to achieve those outcomes (ie, their health care preferences).^13^ Patients also identify their top health priority, namely the health problem they most want to focus on to achieve their health outcome goals.^17^ Clinicians use these priorities-oriented discussions and decisional strategies to align their health care decisions with patients’ priorities.^15,16^ Previous studies of PPC indicated that the PPC framework was feasible and acceptable to patients and clinicians.^14,18,19^ Most participants have been able to identify the specific, actionable, and realistic health outcome goals needed to inform decision-making.^9^ Furthermore, patient priorities–aligned care has been associated with increased priorities-concordant care and decreased treatment burden and unwanted health care compared with usual care (UC) for older adults with MCCs.^16,20^

The current study involved implementation of PPC in a setting outside that of the developers, providing additional evidence of both previous and new outcomes. The aim was to evaluate the association between receiving PPC or UC and patient-reported outcomes (PROs) and days not at home because of health. Because the COVID-19 pandemic hampered enrollment and intervention implementation, we completed the study with modifications.

## Methods

### Study Design and Setting

The study followed a nonrandomized controlled trial design.^21^ The trial protocol is shown in Supplement 1. One primary care site within Cleveland Clinic’s multisite Primary Care Practice was selected as the PPC site (Lakewood, Ohio). The UC site (Brunswick, Ohio) was identified as the optimal match to the PPC site using a multivariate matching procedure that calculated the multivariate distance between the PPC site and 11 potential UC sites.^22,23^ Matching variables included the percentage of patients who were aged 65 years or older, aged 65 years or older and any race other than White, and aged 65 years or older and dual Medicare-Medicaid recipients. The distance function accounts for variances and correlations of the variables across all sites. Additional details about the methods are shown in the eAppendix in Supplement 2.

The Cleveland Clinic institutional review board approved the study. Oral consent was obtained from study participants. This article follows Transparent Reporting of Evaluations With Nonrandomized Designs (TREND) reporting guidelines for nonrandomized trials.^24^

### Participants and Enrollment

#### Clinicians

Six of the 7 primary care practitioners (PCPs) providing care at the PPC site participated, including 4 physicians and 2 advanced practice practitioners (APPs). A PPC site physician (H.N.), served as clinical champion, supporting his colleagues in implementing PPC. One PCP, who was retiring, declined. All 9 PCPs (6 physicians and 3 APPs) at the UC site participated.

#### Patients

Potentially eligible patients were those cared for by the participating PCPs. Inclusion criteria included age 65 years or older and 3 or more chronic conditions plus any of 10 or more medications; 2 or more specialist visits, more than 2 emergency department (ED) visits or more than 1 hospitalization or 10 or more hospital days; or received care coordination services, in the past year. Exclusion criteria included not speaking English, meeting hospice criteria, advanced dementia or moderate to profound intellectual disabilities, or long-term nursing home (NH) resident. Administrative data were used to identify patients of the participating PCPs meeting these criteria who had a scheduled visit within 6 weeks. Potential participants were invited through the electronic health record (EHR) if they had an active patient portal or by letter if not. They then were contacted by telephone to explain the project, determine final eligibility (primarily cognition, very advanced illness, language, NH residence), and obtain consent. Eligible and consenting participants cared for by UC PCPs completed the baseline interview (described later) during this call as did PPC participants initially. The protocol was modified so that health priorities identification occurred during the first call; the baseline interview could occur that day or be scheduled for another day for PPC participants. The enrollment date was the date of the baseline interview except for PPC participants who declined the baseline interview but completed priorities identification; enrollment began with priorities identification for these latter participants. Enrollment occurred between August 21, 2020, and May 14, 2021. Follow-up continued through February 26, 2022. The flow diagram of PPC and UC participants is displayed in the Figure.

**Figure.**
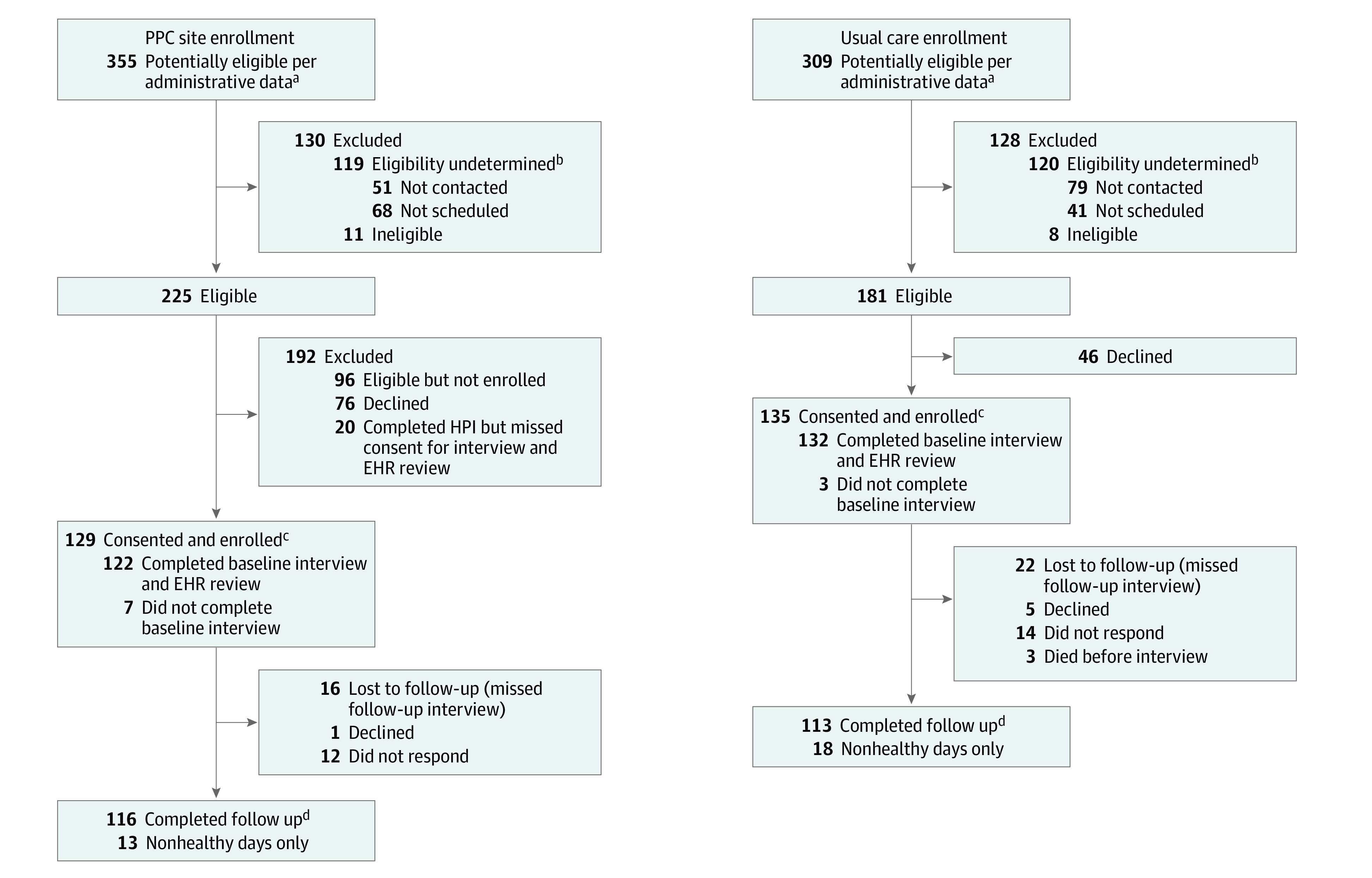
Participant Flow Diagram EHR indicates electronic health record; HPI, health priorities identification; PPC, patient priorities care. ^a^Preliminary eligibility was based on administrative data and included age 65 years or older and 3 or more chronic conditions, plus any of 2 or more specialist visits past year, more than 2 emergency department visits or more than 1 hospitalization or 10 or more hospital days in past year; English speaking; not meeting hospice criteria; no advanced dementia diagnosis; and not a long-term nursing home resident. ^b^Refers to patients who were potentially eligible according to administrative data but were never scheduled for screening call to verify eligibility or were scheduled but canceled and not rescheduled, often because of staff deployments because of COVID-19. ^c^Enrollment for PPC participants required health priorities identification with or without baseline interview or EHR review. Enrollment for usual care participants required baseline interview or agreement with EHR review. ^d^Complete follow-up required both follow-up interview and nonhealthy days.

### PPC Intervention

The development of PPC has been described previously.^13,14,15,16^ We used a practice change framework (eg, leadership support, clinical champions, training, workflow support, health information technology enhancements, and collaborative learning) and plan-do-study-act cycles to implement PPC.^25,26,27^ Four members of the health care team (an APP, nurse navigator, care coordinator, and geriatrician) completed previously described training to become health priorities facilitators^13,16^ During telephone visits, the facilitators guided patients, and caregivers when desired, through identification of the following: (1) values; (2) specific, actionable, and realistic outcome goals; (3) health care preferences (eg, medications, health care visits, tests, procedures, self-management tasks, supportive services they felt either were doable and helpful or did not help or were bothersome); and (4) the top priority (ie, health problem) they most wanted their clinicians to focus on because it was bothersome or impeding achievement of their most desired outcome goal.^13,16,17^ The completed patient health priorities template, including this information, was placed in an accessible location in the EHR.

Participating PCPs at the PPC site completed a 2-hour virtual training, led by the PPC investigator team (M.T., J.E., A.N., and L.D.), based on previously described PPC training.^15,16^ The PCPs and health priorities facilitators then piloted PPC for 2 months before enrollment began. The PCPs participated in eight 15- to 30-minute, case-based huddles over 12 months, facilitated by the Cleveland Clinic PPC site principal investigator (A.H.) and clinical champion (H.N.) and attended by members of the PPC team (M.T., J.E., A.N., and L.D.).^28^ PCPs were alerted to the presence of their patient’s health priorities template in the EHR. For all visits, PCPs were instructed to discuss the priorities and use patient priorities decisional strategies (ie, use patients’ health priorities as focus of communication and decision-making; use serial trials to start, stop, or continue care based on achieving health outcome goals and consistent with health care preferences; and align decision-making among clinicians when there are different perspectives or recommendations) to decide with patients what care to stop, start, or continue.^15,29^ PPC decisional guidance also included strategies for troubleshooting challenges such as when patients’ health outcome goals were not achievable either because of health status or because patients were not able or willing to receive the health care necessary to achieve their outcome goals.^30^

### Outcomes

The primary PROs included (1) perceived treatment burden measured by the Treatment Burden Questionnaire (TBQ) (score range, 0-150, with higher score denoting greater perceived treatment burden; Cronbach α = 0.90)^31^; (2) achievement of desired activities measured by the Patient-Reported Outcomes Measurement Information System (PROMIS) Ability to Participate in Social Roles and Activities Short Form 6a (score range, 6-30, with higher score denoting more social roles or activities; Cronbach α = 0.98)^32^; and (3) CollaboRATE (Cronbach α = 0.89; score range, 0-100 with higher score denoting greater perceived shared decision-making; dichotomized as 100 vs <100, with 100 being the percentage of participants who reported the top score of all 3 items).^33^ The secondary outcome was the Cleveland Clinic Accountable Care Organization (ACO) shared prescribing decision-making quality measure. The PROs were ascertained by phone at baseline and after 9 months of follow-up; PROMIS Social Roles and Activities was ascertained only at the 9-month follow-up.

Nonhealthy days, a primary outcome, was a modified inverse of the healthy days at home measure, similar to health care contact days.^34,35,36,37^ Nonhealthy days were the number of days in which persons were dead or in the hospital, ED, NH, or undergoing ambulatory procedures requiring several hours for completion and recovery (eg, endoscopy or ambulatory surgical procedures). Persons were included in only 1 category per day (in the following order: death, hospital, ED, procedure, or NH). These data were ascertained from EHR covering 90 days before and 365 days after enrollment. CollaboRATE and TBQ were included in the previous studies; the other outcomes were not.^16^ Outcomes were ascertained by assessors blinded to site; data entry forms did not include PCP site.

### Covariates

Sociodemographic data, 18 chronic conditions (based on Centers for Medicare and Medicaid Services’ Chronic Conditions Warehouse *International Statistical Classification of Diseases and Related Health Problems, Tenth Revision *algorithms), and medications were ascertained from the EHR.^38^ During the baseline interview, memory was assessed with 5-item recall.^39^ The physical and mental health measures from the PROMIS global were included in the baseline and follow-up interviews.^40^ Baseline characteristics included in the propensity score included age, sex, self-reported race (eg, Asian or Pacific Islander, Black or African American, Native American or Native Alaskan, and White), living situation, education, health insurance, number of chronic conditions and medications, presence of heart failure or chronic lung disease, 5-item recall, baseline physical and mental health, treatment burden, CollaboRATE, ACO shared prescribing decision-making quality measure, and nonhealthy days in the 3 months prior to enrollment. Information on race was included in this study as a descriptive characteristic of the study population.

### Effects of COVID-19 on Execution of the Study

Several protocol challenges resulted from the COVID-19 pandemic. First, training was moved from in-person to virtual. A delay of several months in the start of enrollment and anticipated deployments of PCPs and team members to COVID-19–related duties resulted in a change in targeted enrollment from 500 to 250. Actual COVID-19–related deployments led to missing potentially eligible participants (Figure). Eligible PPC participants missed enrollment either because the consent visit could not be scheduled (20 participants) or because they missed the baseline interview when we modified the protocol to allow health priorities identification to occur during the first encounter (7 participants). Patients received multiple check-in telephone calls from Cleveland Clinic teams, and some patients expressed difficulty distinguishing the health priorities call from other calls; in addition, facilitators and baseline interviewers were working at home and calling from their private lines, resulting in fewer calls answered owing to unfamiliar numbers on the caller identification. Finally, PCP appointments were rescheduled during COVID-19 surges, leading to temporal gaps between priorities identification and PCP visits and deferred care for chronic conditions.

### Statistical Analysis

Data analysis was performed from March 2022 to August 2023. Missing data were handled by using multiple imputation using the fully conditional specification procedure in SAS statistical software version 9.4 (SAS Institute).^41^ Those who died during the study prior to follow-up were assigned the worst score for the outcome prior to imputation.

We estimated propensity scores using logistic regression for each imputed data set with the PSMATCH procedure in SAS. We used inverse probability weighting to ensure balance in measured patient-level characteristics across groups.^42,43,44^ Weighted covariate balance was evaluated using absolute standardized mean differences of 0.25 or less.^45^

Patient-level variables included in the propensity score model were potential confounders that differ between sites and potentially correlated with the outcomes. For the imputation model, we included all variables used in the propensity analyses, all outcomes, and site.

For the raw data, we compared the distribution of key covariates across site using χ^2^ or *t* tests as appropriate. Statistical significance was defined as 2-sided *P* < .05. We assessed the association of site and key covariates in the weighted and imputed data in models with covariate as the dependent variable and site as the independent variable, within a linear or logistic regression model as appropriate. Propensity score–weighted multivariable linear regression models were used to examine the magnitude of the association between PCP site and PROs and nonhealthy days at baseline and follow-up. The dichotomized CollaboRATE scale and shared prescribing decision-making measure were analyzed using propensity score–weighted logistic regression. All outcome models were also adjusted for the corresponding baseline values for demographic and clinical characteristics.^46^ Robust sandwich variance was used for analysis of complete data. The Rubin formula was used to combine estimates from multiply imputed full data sets into a single set of results using the MIANALYZE procedure in SAS.^41^

## Results

A total of 264 individuals participated, including 129 in the PPC group (mean [SD] age, 75.3 [6.1] years; 66 women [48.9%]) and 135 in the UC group (mean [SD] age, 75.6 [6.5] years; 55 women [42.6%]). Participant enrollment and follow-up are shown in the Figure. Of 181 known eligible UC patients, 135 (74.5%) consented and enrolled. A lower percentage of PPC than UC participants were White or attended college (Table 1). Word recall scores were lower and treatment burden scores higher in PPC than UC participants. Baseline characteristics were balanced between groups after imputation and propensity weighting (Table 1, Table 2, and Table 3).

**Table 1.** Baseline Characteristics of Participants

Characteristic	Participants, No. (%)		Unadjusted *P* value	Adjusted *P* value^b^
	Usual care (n = 135)^a^	PPC (n = 129)^a^		
Age, mean (SD), y	75.3 (6.1)	75.6 (6.5)	.73	.90
Sex				
Female	66 (48.9)	55 (42.6)	.31	.84
Male	69 (51.1)	74 (57.4)		
Self-reported race				
Asian or Pacific Islander	2 (1.5)	3 (2.3)	.01^c^	.80^c^
Black or African American	1 (0.8)	10 (7.8)		
Native American or Native Alaskan	0	1 (0.8)		
White	129 (95.6)	110 (85.3)		
Education high school or less	46 (34.8)	24 (19.0)	<.001	.56
Medicare Advantage	63 (46.7)	61 (47.7)	.87	.42
Lives alone	40 (30.3)	54 (43.5)	.03	.68
5-Word recall score, mean (SD)	3.7 (1.4)	3.4 (1.4)	.07	.86
PROMIS physical health, mean (SD)	14.2 (2.8)	14.7 (3.1)	.16	.94
PROMIS mental health, mean (SD)	13.9 (2.8)	14.3 (3.0)	.35	.79
No. of chronic conditions, mean (SD)	6.1 (2.1)	6.0 (2.3)	.92	.93
Heart failure	19 (14.1)	17 (13.2)	.83	.90
Chronic obstructive pulmonary disease	29 (21.5)	36 (27.9)	.23	.68
No. of oral prescription medications for chronic conditions, mean (SD)	8.0 (3.0)	8.5 (3.9)	.24	.58

Abbreviations: PPC, patient priorities care; PROMIS, Patient-Reported Outcomes Measurement Information System.

^a^ Some numbers may not sum to group total because of missing data.

^b^ The analysis data set was imputed and propensity weighted. The adjusted *P* values reflect robust errors. Variables used in propensity weighting included race, sex, education, and baseline measures of age, living alone, insurance, cognitive status, number of oral prescription medications, number of chronic conditions, heart failure, chronic obstructive pulmonary disease, PROMIS mental and physical health, Treatment Burden Questionnaire score, CollaboRATE Score, accountable care organization prescription quality measures, and nonhealthy days in the 3 months before enrollment. In addition to variables used in propensity models, imputation models included practice site (PPC or usual care) and follow-up measures of PROMIS mental and physical health, Treatment Burden Questionnaire score, CollaboRATE Score, accountable care organization prescription quality measure, and number of nonhealthy days during the year following enrollment.

^c^ *P* values are for the comparison of White vs all other categories.

**Table 2.** ORs for Association of Receiving PPC With Patient-Reported Outcomes

Outcome	Participants, weighted % (95% CI)^a^		OR (95% CI)	*P* value
	PPC	Usual care		
CollaboRate Top Score				
Baseline^b^	46.3 (36.5-56.5)	47.7 (37.9-57.8)	0.90 (0.50-1.70)	.84
Follow-up	58.5 (45.6-70.3)	61.9 (50.9-71.8)	0.90 (0.40-1.80)	.69
Accountable care organization shared prescribing decision-making quality measure				
Baseline	48.1 (38.2-58.2)	50.3 (40.4-60.2)	0.90 (0.50-1.60)	.75
Follow-up	72.5 (56.4-84.3)	52.1 (36.7-67.2)	2.40 (0.90-6.40)	.07

Abbreviations: OR, odds ratio; PPC, patient priorities care.

^a^ A higher percentage reflects better outcomes for both measures. All results reflect doubly robust imputed models. Variables used in propensity weighting included race, sex, education, and baseline measures of age, living alone, insurance, cognitive status, number of oral prescription medications, number of chronic conditions, heart failure, chronic obstructive pulmonary disease, baseline Patient-Reported Outcomes Measurement Information System (PROMIS) mental health and physical health, baseline Treatment Burden Questionnaire score, baseline CollaboRATE Score, baseline accountable care organization shared prescribing decision-making quality measure, and nonhealthy days in 90 days before enrollment. In addition to variables used in propensity models, imputation models included practice site and follow-up measures of PROMIS mental health, PROMIS physical health, Treatment Burden Questionnaire score, CollaboRATE Score, accountable care organization shared prescribing decision-making quality measure as well as number of nonhealthy days during the year following enrollment. Covariates in outcome models included race, sex, education, and baseline measures of age, living alone, cognitive status, number of oral prescription medications, number of chronic conditions, heart failure, chronic obstructive pulmonary disease, and baseline PROMIS mental health and physical health.

^b^ The baseline outcome is included to illustrate weighted balance of covariate before PPC is introduced, and the *P* values for the baseline outcome comparison are based on weighted and imputed data (the same as the adjusted *P* values in Table 1). Of note, the baseline value of the outcome was included in outcome models at follow-up. Therefore, the difference between estimates for baseline and follow-up is not necessarily equal to the effect size reported.

**Table 3.** Differences Between Receiving PPC and Patient-Reported Outcomes^a^

Outcome	Score, mean (95% CI)		Difference, mean (95% CI)	*P* value
	PPC	Usual care		
TBQ scale^b^^,^^c^				
Baseline	11.9 (10.3 to 13.6)	11.6 (9.3 to 14.0)	0.3 (−2.6 to 3.2)	.84
Follow-up	12.7 (10.0 to 15.5)	17.9 (13.1 to 22.7)	−5.2 (−10.9 to −0.5)	.07
PROMIS social roles and activities^d^				
Baseline	NA	NA	NA	NA
Follow-up	21.9 (20.6 to 23.1)	21.6 (20.2 to 22.9)	0.3 (−1.6 to −2.2)	.75
Nonhealthy days^e^				
Baseline	0.7 (0.4 to 1.0)	0.7 (0.4 to 1.0)	−0.0 (−0.4 to 0.4)	.85
Follow-up	8.7 (3.8 to 13.7)	13.4 (6.6 to 20.1)	−4.6 (−12.9 to −3.6)	.27

Abbreviations: NA, not applicable; PPC, patient priorities care; PROMIS, Patient-Reported Outcomes Measurement Information System; TBQ, Treatment Burden Questionnaire.

^a^ A lower score is better for TBQ and nonhealthy days; a higher score is better for the PROMIS measure. All results reflect doubly robust imputed models. Variables used in propensity weighting included race, sex, education, and baseline measures of age, living alone, insurance, cognitive status, number of oral prescription medications, number of chronic conditions, heart failure, chronic obstructive pulmonary disease, baseline PROMIS mental health and physical health, baseline TBQ score, baseline CollaboRATE Score, baseline accountable care organization shared prescribing decision-making quality measure, and nonhealthy days in 90 days before enrollment. In addition to variables used in propensity models, imputation models included practice site and follow-up measures of PROMIS mental health, PROMIS physical health, TBQ score, CollaboRATE Score, accountable care organization shared prescribing decision-making quality measure as well as number of nonhealthy days during the year following enrollment. Covariates in outcome models included race, sex, education, and baseline measures of age, living alone, cognitive status, number of oral prescription medications, number of chronic conditions, heart failure, chronic obstructive pulmonary disease, and baseline PROMIS mental health and physical health.

^b^ The baseline outcome is included to illustrate weighted balance of covariate before PPC is introduced, and the *P* values for the baseline outcome comparison are based on weighted and imputed data (same as the adjusted *P* values in Table 1). Of note, the baseline value of the outcome was included in outcome models at follow-up. Therefore, the difference between estimates for baseline and follow-up is not necessarily equal to the effect size reported.

^c^ Lower score reflects less perceived treatment burden.

^d^ Higher score reflects more social participation. PROMIS Social Roles and Activities was inadvertently left out of the baseline interview.

^e^ Baseline refers to 90 days before enrollment; follow-up refers to 365 days after enrollment. Baseline measures of nonhealthy days include only encounter days.

For PPC participants, the median (IQR) time between identification of health priorities and first PCP visit was 7 (1-11) days. Of 129 PPC participants, 116 (89.9%) completed the follow-up interview, as did 113 of 135 (83.7%) UC participants. All participants had complete nonhealthy days data. During 1-year follow-up, 2 PPC and 5 UC participants died.

In response to the shared prescribing decision-making measure, a higher percentage of PPC than UC participants reported that their clinician asked them what they thought was best (weighted proportions, 72.5% [95% CI, 56.4%-84.3%] vs 52.1% [95% CI, 36.7%-67.2%]), and PPC participants were almost 2.5 times more likely than UC participants to endorse shared prescribing decision-making (odds ratio, 2.40; 95% CI, 0.90-6.40; *P* = .07), although the difference was not statistically significant (Table 2). The proportions reporting top CollaboRATE score were similar in the 2 groups at follow-up.

There was no statistically significant difference in perceived treatment burden score between groups in multivariate models (least square means difference, −5.2 points; 95% CI, −10.9 to −0.5 points; *P* = .07) (Table 3). Participants in the PPC group experienced 4.6 fewer nonhealthy days (95% CI, −12.9 to −3.6 days; *P* = .27) compared with the UC participants over 1-year follow-up, although the difference was not statistically significant. There was no difference between groups in the social roles and activities measure.

## Discussion

Although the findings of this nonrandomized controlled trial did not meet statistical significance, the group who received care aligned with patients’ health priorities did have better scores for treatment burden and shared prescribing decision-making. The former remained relatively stable in PPC participants while increasing among UC participants. In an earlier study, PPC participants reported significantly improved treatment burden at follow-up.^16^ At follow-up, patients from the PPC practice were 2.40 times more likely to report shared prescribing decision-making than patients from the UC practice. The importance of the statistically nonsignificant, but potentially meaningful, 4.6 fewer nonhealthy days among PPC than UC participants during follow-up is unclear and requires exploration in larger confirmatory trials. PROMIS Social Roles and Activities were similar in the 2 groups at follow-up. CollaboRATE scores did not differ between the groups, similar to our previous study.^16^ Ubbink et al^47^ suggested that high CollaboRATE scores for some individuals may signify general satisfaction rather than shared decision-making.

Goal and priorities–directed care for persons with MCCs has been studied by other investigators with mixed results.^48,49,50,51^ Three recent systematic reviews^48,49,50^ of collaborative goal setting or shared decision-making among persons with MCCs found no difference in health-related quality of life or health care utilization and minimal effect on patient satisfaction or caregiver burden. Intervention participants were more likely to perceive that care was related to their goals, and there was greater inclusion of goals in care plans in some studies.^49,50^ A small feasibility study^51^ found goal attainment scaling to be feasible for use in primary care with patients with MCCs. To our knowledge, no prior study, other than our earlier trial,^16^ included interventions aimed at preparing both patients and clinicians in goals and priorities identification and aligning care with these priorities while studying a range of patient reported and health care utilization outcomes.

### Limitations and Strengths

This nonrandomized study has several limitations. Although the UC site was the optimal match to the PPC site and propensity-adjusted samples were balanced, we cannot eliminate the possibility that unmeasured confounders explain outcome differences. Similarly, multiple imputation cannot address bias due to nonmissing at random. Disparity in proportion of persons from minoritized racial groups between the groups occurred despite being a site selection criterion and oversampling, highlighting the ongoing challenges in recruiting minoritized populations.^52^ The single site and small numbers further preclude generalization, although corroboration of some previous results is promising.^16^

We did not adjust for multiple primary outcomes. First, this nonrandomized trial is largely exploratory, aimed at providing evidence to support future, definitive randomized trials. Second, the outcomes represent distinct aspects of patient care, as recommended for persons with MCCs.^53,54^ Hence, results are reported as separate conclusions. Opinions and practices remain mixed as to whether adjustment for multiple testing should be made in pragmatic trials not seeking marketing authorization.^55^

As for many studies, the COVID-19 pandemic resulted in delays, disruptions, protocol deviations, and reduced sample size, the latter reducing power to detect statistically significant differences for promising outcomes. Constraints in sample size rendered analyses aimed more at describing promising trends. We cannot determine what might have happened in the absence of COVID-19 and with the larger planned sample size. We did not calculate post hoc power, because such analysis is often discouraged.^56,57^ In addition, eligible PPC participants either missed enrollment because the consent visit could not be scheduled (20 participants) or because they missed the baseline interview when we modified the protocol to allow health priorities identification to occur during the first encounter (7 participants). Furthermore, follow-up was limited to 9 months (12 months for the utilization-based nonhealthy days).

Despite these limitations, important strengths remain. As has been recommended, we prepared both clinicians and patients to align decision-making with patients’ health priorities, developed a systematic approach to identifying patients’ health priorities, incorporated strategies for clinicians to translate these priorities into care options and align decisions with them, provided suggestions for incorporating PPC into the busy clinical workflow, and investigated effects on patients, clinicians, and the health care system.^53^ The study included PROs and health care utilization, which are recommended to assess values and outcomes for age-friendly care.^54^ Nonhealthy days, like its inverse, healthy days at home, is a measure based on health care utilization that is of importance to patients, health systems, and payers.^34,35,36^ Given its meaningfulness, this measure deserves further study. Response to the ACO shared prescribing decision-making question further suggests that PPC may offer value for patients and health systems because it was part of the quality criterion upon which shared savings reimbursement was based.

## Conclusions

Research in larger and more diverse settings is needed, preferably using participant or site randomization. Our current findings in concert with earlier results, however, suggest that knowing and acting on the priorities of older adults with MCCs may improve some patient outcomes. With evidence in larger samples, patient priorities–aligned decision-making may prove to be a value-based approach for older adults with MCCs who often receive health care that is of uncertain benefit, potential harm and burden, and not focused on their health priorities.

## Notes

Supplement 1.
Trial Protocol and Statistical Analysis Plan

Supplement 2. **eAppendix.** Supplemental Methods
eReferences

Supplement 3.
Data Sharing Statement

## References

[1] Buttorff C, Teague R, Bauman M. Multiple chronic conditions in the United States. RAND Corporation. 2017. Accessed August 14, 2023. https://www.rand.org/pubs/tools/TL221.html

[2] Boyd C, Smith CD, Masoudi F, . Decision making for older adults with multiple chronic conditions: executive summary for the American Geriatrics Society guiding principles on the care of older adults with multimorbidity. J Am Geriatr Soc. 2019;67(4):665-673. doi:10.1111/jgs.15809 30663782

[3] Boyd CM, Wolff JL, Giovannetti E, . Healthcare task difficulty among older adults with multimorbidity. Med Care. 2014;52(3)(suppl 3):S118-S125. doi:10.1097/MLR.0b013e3182a977da 24561750 PMC3937858

[4] Jowsey T, Yen L, W PM. Time spent on health related activities associated with chronic illness: a scoping literature review. BMC Public Health. 2012;12(1):1044. doi:10.1186/1471-2458-12-1044 23206340 PMC3533987

[5] Lorgunpai SJ, Grammas M, Lee DSH, McAvay G, Charpentier P, Tinetti ME. Potential therapeutic competition in community-living older adults in the U.S.: use of medications that may adversely affect a coexisting condition. PLoS One. 2014;9(2):e89447. doi:10.1371/journal.pone.0089447 24586786 PMC3934884

[6] Fried TR, Tinetti ME, Iannone L, O’Leary JR, Towle V, Van Ness PH. Health outcome prioritization as a tool for decision making among older persons with multiple chronic conditions. Arch Intern Med. 2011;171(20):1854-1856. doi:10.1001/archinternmed.2011.424 21949032 PMC4036681

[7] Fried TR, Tinetti ME, Agostini JV, Iannone L, Towle V. Health outcome prioritization to elicit preferences of older persons with multiple health conditions. Patient Educ Couns. 2011;83(2):278-282. doi:10.1016/j.pec.2010.04.032 20570078 PMC2945432

[8] Fried TR, Tinetti ME, Towle V, O’Leary JR, Iannone L. Effects of benefits and harms on older persons’ willingness to take medication for primary cardiovascular prevention. Arch Intern Med. 2011;171(10):923-928. doi:10.1001/archinternmed.2011.32 21357797 PMC3101287

[9] Tinetti ME, Costello DM, Naik AD, . Outcome goals and health care preferences of older adults with multiple chronic conditions. JAMA Netw Open. 2021;4(3):e211271. doi:10.1001/jamanetworkopen.2021.1271 33760091 PMC7991967

[10] Clair CA, Henry M, Jennings LA, Reuben DB, Sandberg SF, Giovannetti ER. Refining a taxonomy of goals for older adults with functional limitations and their caregivers to inform care planning J Appl Gerontol. 2021;40(9):1008-1019. doi:10.1177/0733464820944326 32720843

[11] Tinetti ME, Esterson J, Ferris R, Posner P, Blaum CS. Patient priority-directed decision making and care for older adults with multiple chronic conditions. Clin Geriatr Med. 2016;32(2):261-275. doi:10.1016/j.cger.2016.01.012 27113145

[12] Ferris R, Blaum C, Kiwak E, . Perspectives of patients, clinicians, and health system leaders on changes needed to improve the health care and outcomes of older adults with multiple chronic conditions. J Aging Health. 2018;30(5):778-799. doi:10.1177/0898264317691166 28553806

[13] Naik AD, Dindo LN, Van Liew JR, . Development of a clinically feasible process for identifying individual health priorities. J Am Geriatr Soc. 2018;66(10):1872-1879. doi:10.1111/jgs.15437 30281794 PMC10185433

[14] Blaum CS, Rosen J, Naik AD, . Feasibility of implementing patient priorities care for older adults with multiple chronic conditions. J Am Geriatr Soc. 2018;66(10):2009-2016. doi:10.1111/jgs.15465 30281777 PMC7015118

[15] Tinetti M, Dindo L, Smith CD, . Challenges and strategies in patients’ health priorities-aligned decision-making for older adults with multiple chronic conditions. PLoS One. 2019;14(6):e0218249. doi:10.1371/journal.pone.0218249 31181117 PMC6557523

[16] Tinetti ME, Naik AD, Dindo L, . Association of patient priorities-aligned decision-making with patient outcomes and ambulatory health care burden among older adults with multiple chronic conditions: a nonrandomized clinical trial. JAMA Int Med. 2019;179(12):1688-1697. doi:10.1001/jamainternmed.2019.4235 31589281 PMC6784811

[17] Davenport C, Ouellet J, Tinetti ME. Use of the patient-identified top health priority in care decision-making for older adults with multiple chronic conditions. JAMA Netw Open. 2021;4(10):e2131496. doi:10.1001/jamanetworkopen.2021.31496 34709390 PMC8554637

[18] Feder SL, Kiwak E, Costello D, . Perspectives of patients in identifying their values-based health priorities. J Am Geriatr Soc. 2019;67(7):1379-1385. doi:10.1111/jgs.15850 30844080 PMC6612577

[19] Ouellet GM, Kiwak E, Costello DM, . Clinician perspectives on incorporating patients’ values-based health priorities in decision-making. J Am Geriatr Soc. 2021;69(1):267-269. doi:10.1111/jgs.16914 33165913 PMC7839399

[20] Freytag J, Dindo L, Catic A, . Feasibility of clinicians aligning health care with patient priorities in geriatrics ambulatory care. J Am Geriatr Soc. 2020;68(9):2112-2116. doi:10.1111/jgs.16662 32687218

[21] Handley MA, Lyles CR, McCulloch C, Cattamanchi A. Selecting and improving quasi-experimental designs in effectiveness and implementation research. Annu Rev Public Health. 2018;39:5-25. doi:10.1146/annurev-publhealth-040617-014128 29328873 PMC8011057

[22] Rubin DB. Using multivariate matched sampling and regression adjustment to control bias in observational studies. J Am Stat Assoc. 1979;74:318-328. doi:10.2307/2286330

[23] Zhao Z. Using matching to estimate treatment effects: data requirements, matching metrics, and Monte Carlo evidence. Rev Econ Stat. 2004;86(1):91-107. doi:10.1162/003465304323023705

[24] Haynes AB, Haukoos JS, Dimick JB. TREND reporting guidelines for nonrandomized/quasi-experimental study designs. JAMA Surg. 2021;156(9):879-880. doi:10.1001/jamasurg.2021.0552 33825826

[25] Lau R, Stevenson F, Ong BN, . Achieving change in primary care—causes of the evidence to practice gap: systematic reviews of reviews. Implement Sci. 2016;11(1):40. doi:10.1186/s13012-016-0396-4 27001107 PMC4802575

[26] Noël PH, Lanham HJ, Palmer RF, Leykum LK, Parchman ML. The importance of relational coordination and reciprocal learning for chronic illness care within primary care teams. Health Care Manage Rev. 2013;38(1):20-28. doi:10.1097/HMR.0b013e3182497262 22310483 PMC3383880

[27] Batalden PB, Stoltz PK. A framework for the continual improvement of health care: building and applying professional and improvement knowledge to test changes in daily work. Jt Comm J Qual Improv. 1993;19(10):424-447. doi:10.1016/S1070-3241(16)30025-6 8252125

[28] Ouellet JA, Kiwak E, Tinetti ME, . A qualitative study of coaching patient priorities-aligned decision-making through virtual case-based discussions. J Am Geriatr Soc. Published online October 3, 2023. doi:10.1111/jgs.18609 37787061 PMC11045177

[29] Patient Priorities Care. Patient priorities care decisional guidance. Accessed August 14, 2023. http://decisionguide.patientprioritiescare.org

[30] Patient Priorities Care. Troubleshooting: common challenges in aligning decisions with patients’ health priorities. Accessed August 14, 2023. https://patientprioritiescare.org/decisionguide/troubleshooting

[31] Tran VT, Harrington M, Montori VM, Barnes C, Wicks P, Ravaud P. Adaptation and validation of the Treatment Burden Questionnaire (TBQ) in English using an internet platform. BMC Med. 2014;12:109. doi:10.1186/1741-7015-12-109 24989988 PMC4098922

[32] Hahn EA, Kallen MA, Jensen RE, . Measuring social function in diverse cancer populations: evaluation of measurement equivalence of the Patient Reported Outcomes Measurement Information System^®^ (PROMIS^®^) Ability to Participate in Social Roles and Activities short form. Psychol Test Assess Model. 2016;58(2):403-421. 30221102 PMC6136841

[33] Barr PJ, Forcino RC, Thompson R, . Evaluating CollaboRATE in a clinical setting: analysis of mode effects on scores, response rates and costs of data collection. BMJ Open. 2017;7(3):e014681. doi:10.1136/bmjopen-2016-014681 28341691 PMC5372080

[34] Burke LG, Orav EJ, Zheng J, Jha AK. Healthy days at home: a novel population-based outcome measure. Healthc (Amst). 2020;8(1):100378. doi:10.1016/j.hjdsi.2019.100378 31708403

[35] Groff AC, Colla CH, Lee TH. Days spent at home: a patient-centered goal and outcome. N Engl J Med. 2016;375(17):1610-1612. doi:10.1056/NEJMp1607206 27783911 PMC5996758

[36] Lee H, Shi SM, Kim DH. Home time as a patient-centered outcome in administrative claims data. J Am Geriatr Soc. 2019;67(2):347-351. doi:10.1111/jgs.15705 30578532 PMC6367008

[37] Bynum JPW, Meara ER, Chang CH, Rhoads JM, Bronner KK. Our parents, ourselves: health care for an aging population. A report of the Dartmouth Atlas Project. February 17, 2016. Accessed December 13, 2023. https://data.dartmouthatlas.org/downloads/reports/Our_Parents_Ourselves_021716.pdf 36508510

[38] Chronic Conditions Warehouse. 27 CCW chronic conditions algorithm. Accessed August 15, 2023. https://www2.ccwdata.org/documents/10280/19139421/ccw-chronic-condition-algorithms.pdf

[39] Nasreddine ZS, Phillips NA, Bédirian V, . The Montreal Cognitive Assessment, MoCA: a brief screening tool for mild cognitive impairment. J Am Geriatr Soc. 2005;53(4):695-699. doi:10.1111/j.1532-5415.2005.53221.x 15817019

[40] Hays RD, Bjorner JB, Revicki DA, Spritzer KL, Cella D. Development of physical and mental health summary scores from the patient-reported outcomes measurement information system (PROMIS) global items. Qual Life Res. 2009;18(7):873-880. doi:10.1007/s11136-009-9496-9 19543809 PMC2724630

[41] Rubin DB. Multiple Imputation for Nonresponse in Surveys. Wiley; 1987. doi:10.1002/9780470316696

[42] Stuart EA. Matching methods for causal inference: a review and a look forward. Stat Sci. 2010;25(1):1-21. doi:10.1214/09-STS313 20871802 PMC2943670

[43] Austin PC. An introduction to propensity score methods for reducing the effects of confounding in observational studies. Multivariate Behav Res. 2011;46(3):399-424. doi:10.1080/00273171.2011.568786 21818162 PMC3144483

[44] Austin PC, Stuart EA. Moving towards best practice when using inverse probability of treatment weighting (IPTW) using the propensity score to estimate causal treatment effects in observational studies. Stat Med. 2015;34(28):3661-3679. doi:10.1002/sim.6607 26238958 PMC4626409

[45] Rubin DB. Using propensity scores to help design observational studies: application to the tobacco litigation. Health Serv Outcome Res Methodol. 2001;2:169-188. doi:10.1023/A:1020363010465

[46] Tsiatis AA, Davidian M. Demystifying double robustness: a comparison of alternative strategies for estimating a population mean from incomplete data. Stat Sci. 2007;22(4):569-573. doi:10.1214/07-STS227B 18516239 PMC2397555

[47] Ubbink DT, van Asbeck EV, Aarts JWM, . Comparison of the CollaboRATE and SDM-Q-9 questionnaires to appreciate the patient-reported level of shared decision-making. Patient Educ Couns. 2022;105(7):2475-2479. doi:10.1016/j.pec.2022.03.007 35331573

[48] Butterworth JE, Hays R, McDonagh STJ, . Involving older people with multimorbidity in decision-making about their primary healthcare: a Cochrane systematic review of interventions (abridged). Patient Educ Couns. 2020;103(10):2078-2094. doi:10.1016/j.pec.2020.04.008 32345574

[49] Vermunt NPCA, Harmsen M, Westert GP, Olde Rikkert MGM, Faber MJ. Collaborative goal setting with elderly patients with chronic disease or multimorbidity: a systematic review. BMC Geriatr. 2017;17(1):167. doi:10.1186/s12877-017-0534-0 28760149 PMC5537926

[50] Smith SM, Wallace E, O’Dowd T, Fortin M. Interventions for improving outcomes in patients with multimorbidity in primary care and community settings. Cochrane Database Syst Rev. 2016;3(3):CD006560. doi:10.1002/14651858.CD006560.pub3 26976529 PMC6703144

[51] Ford JA, Lenaghan E, Salter C, . Can goal-setting for patients with multimorbidity improve outcomes in primary care? cluster randomised feasibility trial. BMJ Open. 2019;9(6):e025332. doi:10.1136/bmjopen-2018-025332 31164362 PMC6561432

[52] Rhodes RL, Barrett NJ, Ejem DB, . A review of race and ethnicity in hospice and palliative medicine research: representation matters. J Pain Symptom Manage. 2022;64(5):e289-e299. doi:10.1016/j.jpainsymman.2022.07.009 35905937

[53] Shiner A, Player E, Salter C, Steel N. Goal-setting with patients with multi-morbidity: finding a way to achieve ‘what really matters’. InnovAiT. 2020;13(3):179-185. doi:10.1177/1755738019891190

[54] Burke RE, Ashcraft LE, Manges K, . What matters when it comes to measuring age-friendly health system transformation. J Am Geriatr Soc. 2022;70(10):2775-2785. doi:10.1111/jgs.18002 36053842

[55] Pike K, Reeves BC, Rogers CA. Approaches to multiplicity in publicly funded pragmatic randomised controlled trials: a survey of clinical trials units and a rapid review of published trials. BMC Med Res Methodol. 2022;22(1):39. doi:10.1186/s12874-022-01525-9 35125091 PMC8818238

[56] Hoenig JM, Heisey DM. The abuse of power: the pervasive fallacy of power calculations for data analysis. Am Stat. 2001;55(1):19-24. doi:10.1198/000313001300339897

[57] Dziak JJ, Dierker LC, Abar B. The interpretation of statistical power after the data have been gathered. Curr Psychol. 2020;39(3):870-877. doi:10.1007/s12144-018-0018-1 32523323 PMC7286546

