# Supplementary material for: Patient Priorities–Aligned Care for Older Adults With Multiple Conditions: A Nonrandomized Controlled Trial
Source: JAMA Netw Open. 2024 Jan 23;7(1):e2352666. doi: 10.1001/jamanetworkopen.2023.52666 (PMC10807252; doi:10.1001/jamanetworkopen.2023.52666)
Supplement: Supplement 1. — Trial Protocol and Statistical Analysis Plan [file jamanetwopen-e2352666-s001.pdf]

Introduction

Healthcare for older adults with multiple chronic conditions (MCCs) is burdensome and of uncertain benefit, resulting in unwanted and unhelpful care. Patient Priorities Care (PPC) is an approach that aligns care with patients' health priorities (i.e. the health outcomes most desired given the healthcare each is willing and able to receive). PPC offers the opportunity to increase value by improving both outputs (desired health outcomes) and inputs (healthcare preferences) for these major users of healthcare.

The ultimate goal of our work is to implement and evaluate this approach to care for older adults with multiple chronic conditions that focuses on what matters most to them and is less fragmented and burdensome, resulting in better quality and outcomes at lower cost. This study will focus on evaluating practice change at test sites at the Cleveland Clinic.

Background and Significance

The 60-70% of older adults with multiple chronic conditions (MCCs) are major recipients of healthcare.[1] Much of this healthcare is burdensome and of uncertain or modest benefit.[2] Most evidence is generated in persons with few conditions and assessed by survival or disease-specific outcomes that may not be what matters most to this population who vary in their outcome priorities.[2-8] The burden imposed by caring for MCCs is increasing.[9,10] Medicare patients with MCCs spend an average 33 days per year in contact with the healthcare system and two hours a day on healthcare-related activities.[11,12] Cost to the health system (primarily Medicare) accounts for the bulk of the medical spending among older adults. Out of pocket expenses are growing.[13] Some of this care may be harmful.[14] The uncertain benefit, treatment burden, potential harm, and rising costs all call into question the value of healthcare for older adults with MCCs. True value-based care must be of value to individuals, health systems, and payers.[15] Health systems view value based not only on benefits to the system, but also to the patients they serve. In considering value from a patient's perspective, the personal health outcomes that they hope to achieve – which vary for older adults with MCCs - are the appropriate outputs.[6,16] The inputs include financial costs and indirect costs in terms of the health-related workload.[16] When defined as what patients are willing and able to do, the indirect costs inform healthcare preferences.[17] Patients' desired health outcomes given their healthcare preferences constitute their health priorities.[17-20] Patient Priorities Care aligns healthcare with patients' health priorities.[17-20] We now aim to evaluate its value within the Cleveland Clinic.

Healthcare utilization and costs associated with age and MCCs are well known.[1,21] Persons ≥65 years are 15% of the U.S. population but consume 34% of health care expenditures, including both community-based care and hospitalization.[21,22] Approaches for improving care of older adults with MCCs have been investigated.[21-25] A systematic review of 18 systems- or patient-based interventions - none addressing patients' priorities - revealed modest effect on patient-reported outcomes but little or no effect on

healthcare utilization; cost data were limited.[24,25] Some interventions targeting specific health problems, particularly among persons with advanced illness, have proven cost-effective in the hospital setting; less in known about cost-effectiveness in the ambulatory setting.[26-35]

Compared to patients receiving usual care (UC) in a recent study, those receiving PPC reported greater decrease in treatment burden (a major concern for older adults with MCCs), had more medications stopped and fewer self-management tasks and diagnostic tests ordered.[36]

To determine the value of PPC, comparable primary care sites within the Cleveland Clinic will be assigned to PPC or UC. Clinicians and staff at the PPC site will be trained to identify and align decision-making with the health priorities of older adults with MCCs. Value will be compared using patient and provider-reported outcomes, healthcare utilization, and possibly costs at PPC and UC sites.

Hypothesis/Research Question

Does aligning healthcare with patients' health priorities (their most desired health outcomes given the healthcare activities they are willing and able to do) increase value?

Study Design

Methods

We will employ a quasi-experimental, usual care (UC) group design, involving 2 primary care sites (1 PPC and 1 UC. Patients are assigned to intervention or usual care arms based on their primary care practice location. We will use analytic techniques (e.g., inverse propensity score weighting) designed to reduce selection bias and balance PPC and UC sites in terms of baseline characteristics. Data collection will occur through quantitative and qualitative interviews and health encounter information in the EHR.

Patient Priorities Care requires the elicitation and documentation of patient health outcome goals and care preferences and the alignment of clinical care with goals and priorities to achieve patients' health outcome goals and reduce the burden of multi-morbidity. Participants will be enrolled in the Patient Priorities Care Program and speak with a trained health priorities facilitator to elicit their healthcare preferences and health outcome goals, which together constitute their health priorities. This information will be documented, entered into the EHR, and shared with the clinicians who will then use the Patient Priorities Care approach with patients to inform and guide treatment decisions. Patients will participate in the program and be followed for up to one year from the health priorities identification visit.

<u>Sample</u>

Patients: Potentially eligible participants for this project include Medicare beneficiaries (Traditional, Medicare Advantage, and dual eligible) 66 years or older who are patients of participating clinicians.

We assessed the power to detect clinically relevant differences in Patient-Reported Outcomes (PROs) and categories of healthcare utilization, both needed to measure value. We calculated minimal detectable differences for the primary PPC outcomes based on a sample size of 500 participants (250 PPC and 250 in UC) that assumed 80% power, a one-tailed alpha of 0.05, and an R-squared of 0.2 from other covariates. Assuming a standard deviation of 24.1 at pre-test and 17.6 at post-test and a correlation between measurement pairs of 0.55 (based on pilot data) allows us to detect a between-group difference in mean change in the TBQ score from pre-test to post-test of -4.6 points.[37] Comparing the PPC group to the UC care group, our sample permits us to detect an odds ratio of 1.7 with respect to discontinuing medications and an odds ratio of 0.52 with respect to having diagnostic tests ordered.[38]

<u>Inclusion/Exclusion Criteria</u>

The evaluation of PPC in the clinical practice will take place as part of the patient's ongoing healthcare, and is minimal risk. Criteria for inclusion in the clinical program will be expansive in order to offer the opportunity for the largest number of reachable patients possible. All recruits will be those who are currently patients at Cleveland Clinic. Patient's meeting any of the exclusion criteria will not be approached by the Cleveland Clinic clinicians.

Inclusion Criteria:

1. Age 66 and older
2. In the Cleveland Clinic patient population
3. In the clinician practices selected as intervention or usual care practice sites
4. Clinically identified by: Those who meet any of several criteria
   i. 3 chronic conditions (See appendix 0 for the complete list)
   ii. 10 medications
   iii. >2 ED visits over the past year
   iv. >1 hospitalization (or >10 days in hospital)
   v. receive any care coordination services
   vi. 2 specialists over past year

Exclusion Criteria:

1. In hospice or meeting hospice criteria for any condition
2. Advanced dementia or moderate to profound intellectual disabilities
3. Not English speaking
4. Nursing home resident

Primary Care providers (PCPs) and Health Priorities Facilitators: PCPs are identified at the intervention site by screening the clinic's electronic health records (EHR) to identify those clinicians with eligible patients in their clinic panels. This requires a waiver of consent and waiver of HIPAA authorization. Potential priorities facilitators were identified by examining existing resources and finding most appropriate care givers within the facility (Appendix.1). Employee Study Information sheet will be personally distributed in advance. Research team will directly contact potential clinicians and facilitators, and allow them to review our intervention and expected roles. If potential clinicians and facilitators express interest in the project, they will be also asked to confirm their willingness to complete one hour over-the-phone training session and one half-day training session to be familiar with the intervention process in addition to review of online training materials. At this point, no protected health information or patient identifying information is collected or recorded.

Procedures

The subject for this IRB protocol is the implementation and evaluation of the Patient Priorities Care process that will be implemented in clinical sites at the Cleveland Clinic (CC). The CC Patient Priorities Care team including, primary physicians, and health priorities facilitators (i.e. Geriatrician, Medical Assistant, Care Coordinators, and Patient Liaison Program Coordinators, Nurse practitioners) along with other logistically necessary individuals, will help patients elicit their health outcome goals and healthcare preferences. The elicitation of patient priorities will be embedded into CC clinical programs routinely offered to patients (Appendix 1).

Table 1. Schedule of Evaluation for Patient Population

Clinical Procedures				Goal Facilitation Visit(s)	PPC Clinician Visit(s)	Goal Facilitator Follow up			
Research Procedures		Pre-Screening	Baseline Interview					Follow up interview	Follow up
	Week	Up to -7	-5 to 0	-6 to -1	0	2 to 6 (1-2wk after 1st PCP)		34 to 52	Up to 52
	Month		-1	-1	0	1-2		8 – 12	12
Intervention arm	Consent		x					x	
	EHR data extraction		x						x
	Identification/ discussion of patient health priorities			x	x	x			

	Interview<br>• Demographics<br>• 5-word recall*<br>• PROMIS<br>• CollaboRATE<br>• Tx Burden questionnaire		x					x	
	PCP visit				x				
Usual care arm	Interview<br>• Demographics<br>• 5-word recall*<br>• PROMIS<br>• CollaboRATE<br>• Tx Burden questionnaire		x					x	
	PCP visit				x		X<br>X<br>X		
	EHR data extraction		x						x

*Baseline in-person interview only.

Patients meeting criteria within the Cleveland Clinic Lakewood Family Health Center (FHC) and Brunswick FHC patients who are scheduled to have visit during study period will be recruited using mail, or My Chart message about the project taking place. Lakewood FHC will be serving as intervention site and Brunswick FHC as usual care. Eligible and consented patients will participate in the program and be followed for up to one year from the goal elicitation visit. Primary physicians, and priorities facilitators (i.e. Geriatrician, MAs, Care Coordinators, Patient Liaison Program Coordinators, and Nurse practitioners) will undergo training and preparation to elicit and provide care aligned with health priorities. This training has been developed and tested during the Yale University pilot study of Patient Priorities care.

Pre-screening and Recruitment (Research procedure)

A list of patients meeting above criteria will be generated by the methods described below, and provided to PCPs to obtain their permission to invite patients. PCPs may suggest removing any patients who may not be appropriate for the intervention at PCP's discretion. Research team will conduct a baseline interview after obtaining verbal consent from the patients. Priorities Facilitators will be notified to invite these patients to have their health priorities identified and their care aligned with these priorities. To maximize flexibility and align with current workflow, the invitation for priorities identification can be during a regular visit, by phone, by mail, or other means already used by the practices (e.g. same visit as invitation; part of annual wellness visit, etc.).

Project specific generated list: Each week, a scheduling system will provide an automated list of targeted patients with appointments 6 weeks later. Appointment types include in-person office visit, distance health, virtual, or telephone visit. This system is linked with EHR and widely used at our institution. Once target patients are identified and approved by the PCP, research personnel will contact via mail, or My Chart messaging to introduce the Patient Priorities Care program with an information sheet to explain the Patient Priorities Care process and ask them to contact the research team if they want to opt out. Opt-out patients will be recorded in the screening log to avoid contacting again for future recruitment.

Care providers referral: Care providers can make a referral if they have patients who could benefit from the Patient Priorities Care program, otherwise meet inclusion and exclusion criteria, and have regular visits in next few months. Care providers can discuss the program and provide information sheet to the potential participants. Clinician will notify the research team of the potential patients who have agreed to participate in the PPC. Research team will contact these potential participants to obtain consent to participate in Patient Priorities Care before asking any research questions. Care providers will also notify names of patients who decline to participate in order to avoid contacting the same patients again for future recruitment.

Primary Care Coordination patients list: For patients enrolled through the Care Coordination program, research personnel will identify the individuals ahead of time and obtain permission from their PCPs. Research team will contact via mail, or My Chart messaging to introduce the Patient Priorities Care program with an information sheet to explain the Patient Priorities Care process and ask them to contact research team if they want to opt out. After excluding patients who opted out, research personnel will contact patients to obtain consent and conduct baseline interview. Because this project provides prioritized patients' list based on their existing Care Coordination patient list for the Coordinators to reach out, this process adds very little burden on Care Coordinator's part.

Usual care Group

Concurrent to the Patient Priorities Care implementation, we will begin to identify the usual care group which will include up to 250 patients. We will identify a sample similar to the intervention group, using the same inclusion/exclusion criteria and clinical judgment. The usual care group will be identified from Brunswick FHC patients.

The usual care group will be identified through the same electronic health record search and Care Coordination patient list described above, and PCPs will have the option of not including any of their patients for interview for usual care group.

The usual care group during the study period will receive a recruitment letter from their clinician

describing the research and interview. Patients will be able to opt-out if they do not wish to be contacted by contacting research team listed in the invitation letter. For patients who do not opt-out, a centralized, trained research personnel will contact candidates by phone to confirm eligibility and to obtain verbal consent for enrollment in the usual care arm which involves standard clinical care, data collection through an interview, and a medical record review.

Consent process

We will require a full waiver of consent and HIPAA authorization for the health care utilization data that will be used as part of the screening process and evaluation of the patient population. We will also request a waiver of signed consent and HIPAA authorization for participation of clinicians and patients in the research evaluation. It would be impractical to gain signed consent for this information, specifically for this population and the research involves minimal risk.

Consent process will be conducted primarily over the phone by a trained project staff member. Patients will be notified via letter, My Chart message or phone that a CCF research personnel will be contacting them to conduct the interviews. A trained research coordinator will administer the qualitative survey. Participants in both intervention and usual care groups will receive $10 for each research interview for a total reimbursement of up to $20.

Baseline interview

Once potential patients are identified, research personnel will add participants' contact information into a secure electronic data collection system (REDCap) which the research team will also use to contact the participants to administer the interview. Up to 6 weeks prior to their next PCP appointment, selected patients will be contacted by phone or in-person to confirm consent to participate. Once eligibility is confirmed, research personnel conduct phone or in-person baseline interview whichever is preferred by the patient. Baseline interview could follow after Priorities Facilitators (Geriatrician, MA, Care coordinator, Patient Liaison Program Coordinators, Nurse Practitioners) completes priorities identification process. This can be done before or the day of the initial PCP visit to begin deciding treatment plans based on priorities. Contact information will be stored separately from interview and medical record data and destroyed once the follow-up interviews have been conducted and data collection and cleaning is complete.

Priorities Facilitators will help patients identify their health priorities, complete the health priorities template in EHR. Please note that similar process has been routinely done at the clinic as part of Care Coordinators' and Patient Liaison Program Coordinators' job (Appendix 1), and this project provides more structure in documentation and collaboration with other care providers involved.

PCPs will be asked to review the patients' health priorities at subsequent clinical follow up visits and update if necessary.

At the intervention site, we will identify site champions to develop continued buy-in from providers (e.g., staff meetings, individual meetings, case examples), obtain feedback, and notify the PI and research team of unanticipated problems.

Intervention (Clinical procedure)

Patient Priorities Care is an innovative approach to shared decision-making that draws from existing professional training (e.g. clinical competencies, motivational interviewing, and geriatrics care). Patient Priorities Care requires the elicitation and documentation of patient health outcome goals and care preferences and the alignment of clinical care with health goals and healthcare preferences (collectively referred to as health priorities) Participants will be enrolled in the Patient Priorities Care Program and communicate with a trained priorities facilitator in-person or over the phone to elicit their health priorities. This information will be documented in the PPC- GOALS AND PREFERENCES form in the EHR and shared with the clinicians who will then use the Patient Priorities Care approach with patients to inform and guide treatment decisions.

Patients will participate in the program and be followed for up to one year from the priorities elicitation visit by the research team. The target follow-up time is 8-10 months. The PCPs will be trained in decisional strategies that have been shown to help align care with patients' health priorities. While encouraged to use these decisional strategies, PCPs will be free to make the recommendations they feel most appropriate for each patient.

This intervention has been developed to be integrated seamlessly into usual care, and does not involve any research aspect such as program development, data collection, and evaluation at all. Documentation completed by Facilitators is considered part of standard clinical care. Intervention development, data collection and evaluation including follow up data collection are conducted by the research team. What PCPs and facilitators are asked to perform for this project is within their standard scope of work. The intervention does not require additional visits or substantially increased provider time for PCPs as the Goals and Preferences information collected by the Facilitator will be used as decision aid during regular office visits. However, it is to the providers' discretion, to spend as much time as needed if that would benefit the participating patient.

Priorities Facilitators

The Priorities Facilitators will be trained health professional (i.e. Geriatrician, MA, Care Coordinators, and Patient Liaison Program Coordinators, Nurse practitioners).

Responsibilities

1. Undergo training and preparation to elicit and document patients' health priorities
2. Elicit priorities using PPC point-of-care materials.

3. Provide a copy of completed PPC- GOALS AND PREFERENCES form to the PCP before the PCP visit and encourage patients to bring this up during their PCP visit.
4. Communicate these health priorities to the Primary Care Provider through verbal discussion and/or documentation into the patient's Electronic Health Record (EHR). prior to the PCP visit.
5. Enter the identified health priorities into Electronic Health Record (EHR) and provide to patients (as applicable).
6. Contact enrolled patients to follow up 2-3 weeks after the initial PCP visit and assist in building a partnership between patient and clinician.
7. Review health priorities and update as needed

Clinicians

The clinician will be a licensed health care professional (MD, APRN, PA). Visits can be in-person, virtual, or phone visits.

Responsibilities

1. Participate in health priorities aligned care training that includes a 2 hour face to face session followed by ongoing short review sessions
2. Know or review patient's health priorities template provided by the priorities facilitators, align care decisions to each patient's health priorities "
3. Decision making moves
   a. From: *You need (fill in blank) for your (fill in blank).*
   b. To: *There are different things that we could do. But knowing your conditions, your overall health, and your health outcome goals and care preferences (what matters most to you), I suggest we try (fill in the blank).*
4. Translate patient's health priorities into care options with guidance from point-of-care materials, decisional guidance and strategies, , and clinician champions
5. Participate with patients/caregivers in shared decision-making around health priorities
6. Review, discuss, and update patient's health priorities or refer to priorities facilitator for further discussion
7. Include patient health priorities in clinical communications such as referrals, consults, and clinical notes.
8. Discuss with specialists (e.g. cardiologists) as needed to ensure care is aligned with patient's priorities
9. Document discussions and decisions in EHR (SMART phrases)

Follow up data collection (Research procedure)

Follow up data collection will be two-fold: We'll conduct phone (or in-person) interview and ask the same questions except cognitive ability screening items at 8-10 months from the baseline; and we'll collect patient-level utilization and other clinical variables from EHR from 3 months prior to baseline date through up to 12 months after the baseline date.

Follow up interview: Approximately 9 months from the PCP visit, follow up interview will be conducted by phone or in-person. All patients who participated in the intervention will be contacted at 9 month (+/- 30 days protocol window) to complete follow up interview defined in the variable list. A week prior to follow up interview period, research personnel will send mail/My Chart message or call to remind participants of the follow up interview session. If participant does not respond to the follow up interview invitation by the 9th month, we will follow up with a letter with the interview form and self-addressed, stamped envelope enclosed. We will ask patients to fill the forms and send back by mail.

Post-Follow up interview: Research personnel will contact enrolled patients who completed follow up interview for missed question items from the follow up interview by calling messaging. If an enrolled participant does not respond to the Post-follow up interview invitation by the 12th month from baseline, we will follow up with MyChart message or a letter with the interview form and self-addressed, stamped envelope enclosed. We will ask patients to fill the forms and send back by MyChart message link or mail. This procedure only apply to enrolled patients who completed follow up interview but missed questions due to Redcap programing error reported on 7/15/2021 as Unanticipated Problem.

Data extraction: From 3 months prior to Baseline Interview through up to 1 year from the Baseline Interview, the following information will be ascertained from patients who gave consent: The number and types of chronic conditions (collected at Baseline only), medications, Healthcare activities defined as below, ACP (Advance Care Planning) tab use, and the Accountable Care Organization (ACO) survey as defined below.

Data Collection overview

In the 2 participating sites (Lakewood and Brunswick) the following information will be collected:

- Demographic variables listed in the data collection sheet will be ascertained during the baseline telephone interview or via EHR review. During the baseline interview, physical and mental health will be ascertained with PROMIS-10,[39] while the modified version of cognitive ability screening (5-word memory test) will assess patient'smemory.[40] Number and types of chronic conditions and medications will be ascertained from the EHR.

- In addition to measuring self-reported physical and mental health with PROMIS-10, we will assess patients' perceptions of whether their healthcare was collaborative and focused on their goals with

CollaboRATE.[41] Perceived treatment burden will be assessed by the Treatment Burden Questionnaire.[42] "As part of their ACO quality measures, Cleveland Clinic is focusing on shared decision-making around new medications. We will track responses to the current question on the ACO survey, *"When starting a new medication, did your provider ask what you thought was best for you?"* These PROs will be ascertained at baseline and after approximately 9 months follow-up by a trained assessor.

- The number (%) of participants with the following healthcare activities will be abstracted from EHR orders and visit notes:1) medications; 2) self-management tasks; 3) diagnostic tests 4) referrals; and 5) procedures A data dictionary which guided uniform abstraction in two PPC pilot studies will be used. Inter-rater reliability between two coders was excellent ($\kappa = 0.89$, 95% CI [0.85, 0.95], $p < .001$.[2] Healthcare costs may be estimated by calculating local costs for each activity or service. Hospitalizations and emergency (ED) visits and costs will also be tracked. Time spent in ambulatory healthcare activities, ED visits, and hospital stays will also be used to ascertain healthcare contact days.[43]

- Time required to prepare for, and participate in, patient priorities-aligned decision-making is essential to defining value from the health system perspective. To estimate the time required to implement PPC, research team will work with a process improvement member of PPC practice to build workflows for the tasks needed to carry out PPC by staff and clinicians and calculate the time spent on each task based on interviews and observations. A similar process was used in the PPC pilot study.[44] Time can be converted to dollars using local costs for relevant clinicians and staff.

<u>Clinician feedback</u>

Following the completion of patient recruitment, we will ask each PCP and priorities facilitator for both quantitative and qualitative feedback, including those with both favorable and unfavorable opinions. The quantitative survey was developed and used by investigators implementing PPC in a VA clinic in Texas. We will contact clinicians by email, telephone and in-person, to request their completion of the survey.

COVID-19 Procedures (Remote Research Procedures)

As already described above, entire research procedures including recruitment, consent, baseline interview, Facilitation visit, PCP visits, and follow up interviews, could be performed without direct in-person interactions with participants. The core of the intervention is facilitating discussion among Priorities Facilitator, PCP and patient, regardless of communication modes. We'll examine if there is any mediating effects in results when comparing in-person and phone/virtual communication between patients and care providers.

Data Analysis

We will calculate descriptive statistics for the intervention and usual care participants' baseline characteristics and primary and secondary outcomes. Differences between the two groups' post-test primary and secondary outcomes will be examined using generalized linear models (adjusting for baseline demographic, clinical characteristics and baseline outcome values). Model assumptions will be checked by, for example, inspecting residual plots and goodness-of-fit statistics. To account for clustering of patients within medical practices, we will estimate models with robust standard errors. To minimize the loss of observations used in the analyses, we will use fully-conditional multiple imputation to address missing data. Comparability of participants in the two arms will be assessed by comparing the distribution of baseline characteristics in the two groups using appropriate graphical procedures, summary statistics and multivariable methods. If participants' baseline characteristics appear to be unbalanced between arms, we will use inverse propensity score weighting to achieve covariate balance between the two groups.

Data Confidentiality

Data collection will be performed through the Cleveland Clinic Research Institute, by trained staff, using REDCap, and following Cleveland Clinic's procedures for data protection and privacy. Data will be de-identified before being transmitted to Yale University where it will be prepared for analysis by Yale Program on Aging data core staff.

Chart abstraction of the patient's health record (EHR) occurs at the end of the follow-up period.

All data will be collected and recorded by trained personnel and stored on encrypted computers. PHI will be used only to identify patients eligible for the project. Only Cleveland Clinic clinical staff will have access to the identifiable data. Only de-identified data will be provided to the Yale investigators. At no time will quantitative or qualitative interviews ask for PHI. All data collection, management, and analysis will occur on computers that are encrypted and servers that have appropriate firewalls.

Potential Risks

No more than minimal risk to training participants is expected from this research. For data from electronic health records, there is a potential risk for a breach in confidentiality. All efforts will be made to protect the confidentiality of the participants and their personal health information. We will use study ID numbers for patients so we can link clinical and interview data over the different time points. The data we send to the Yale team will use the study IDs and will not include patient names or medical record numbers.

Protection against Risk

Participant confidentiality will be maintained throughout the project in a way that ensures the information can always be tracked back to the source data. Where appropriate, a unique participant identification code (ID number) will be used that allows identification of all data reported for each participant. Participant information collected in these studies will comply with the standards for protection of privacy of individually identifiable health information as promulgated in the Health Insurance Portability and Accountability Act and as mandated in Title 45 CFR, Parts 160 and 164. All records will be kept confidential and names will not be released by research staff. Caution will be exercised to assure the data are treated confidentially and that provider and patient privacy is protected. To ensure awareness of and compliance with this procedure, all members of the research teams must undergo training on human subject protection and educated regarding the release of PHI for research purposes.

Data will be stored on a password-protected server, which is protected behind a login and Secure Sockets Layer (SSL) encryption. Data will be analyzed using the common programming language and statistical packages. Data access will only be available to the principal investigator and authorized members of the research team.

Electronic record backups are retained in a secure location to prevent catastrophic loss of data quality and integrity and to allow efficient resumption of clinical research following computer failures (see Resources). Electronic audit trials are maintained to protect the authenticity, integrity, and confidentiality. All electronic data systems are behind the Cleveland Clinic firewall. Entry to the continually locked research area is restricted by a coded badge identification system.

The Principal Investigator is ultimately responsible for the conduct of the research protocol. The investigator ensures the research is conducted according to the IRB-approved protocol in compliance with federal regulations, all institutional policies, and ethical principles identified in the Belmont Report. The investigator will ensure that adequate resources are available to conduct the clinical trial and will provide supervision and oversight of all members of the research team. We will also perform data and safety monitoring throughout the study period.

Potential Benefits

The proposed work has potential to directly benefit research participants. Patients will be provided with information that may improve their ability to interact with their clinicians and healthcare team make an informed decisions about their health care. Patient Priorities Care conversations will be integrated as part of clinic standard of care (as part of goals of care conversations). We expect that this approach will create an enhanced dialogue between patients and their clinicians and will result in better care for the individual.

Cost

There is no cost for participation. This quality improvement project is being implementing as part of ongoing patient care at Cleveland Clinic.

Data and Safety Monitoring Plan

Due to the minimal to no-risk nature of the project, the standard of care of these interventions, and the real-time interim data analyses as part of the quality improvement collaborative, a formal Data and Safety Monitoring Board is not necessary. Oversight and monitoring to ensure the safety of participants and the validity and integrity of the data will be ensured by the entire research team, with particular focus by the CCF research team. The research team is committed to protecting participants' privacy throughout the life of the project. At periodic intervals during the course of the project, the research team will:

- Review the research protocol.
- Evaluate the progress of the project.
- Consider factors external to the project when relevant, such as scientific or therapeutic developments that may affect safety or ethics of the project.
- Review performance, make recommendations, and assist in resolving problems.
- Protect the safety of the participants.
- If appropriate, conduct interim analysis
- Ensure confidentiality of data.

Problems with workflow and process will be reviewed by members of the Yale team with the CC research personnel and adjustments will be made as needed. Adverse events will be reported to the IRB as well as to the Yale team and Funders as they occur. Due to the nature of this project, there is minimal risk of adverse events.

Consent

The use of verbal permission is being requested. The research personnel performing the baseline interviews, the health priorities facilitator or the participating clinician will obtain verbal permission from patients who received Patient Priorities Care during the first encounter with patient priorities care. This will allow project staff to provide the pre/post qualitative and quantitative interviews/surveys over the phone but also obtain utilization data. These research personnel, priorities facilitators and clinicians are or will be trained in obtaining verbal permission.
The use of verbal permission will also ensure the safety of potential participants and be appropriate mode of communication during the COVID-19 pandemic.

For initial patient identification process, a waiver of HIPAA authorization is being requested to identify those participating in the clinical program. It would be impractical to gain consent from all the patients whose records will be viewed in order to identify appropriate individuals to invite to participate. These records are being viewed as part of ongoing clinical care, and the information will not be share with any personnel outside of CCF.

References

1. Chevarley, F.M. Expenditures by number of treated chronic condition, race/ethnicity, and age, 2012. Statistical Brief #485. Rockville, MD: Agency for Healthcare Research and Quality; 2015. http://meps.ahrq.gov/mepsweb/data_files/publications/st485/stat485.pdf. Accessed February 21, 2019.
2. Boyd C, Smith CD, Masoudi F, Blaum CS, Dodson J, Green AR, Kelley A, Matlock D, Ouellet J, Rich MW, Schoenborn NL, Tinetti ME. Decision-making for Older Adults with Multiple Chronic Conditions: Executive Summary for the AGS Guiding Principles on the Care of Older Adults with Multimorbidity. J Am Geriatr Soc 2019. doi: 10.1111/jgs.15809.
3. O'Hare AM, Hotchkiss JR, Kurella Tamura M, Larson EB, Hemmelgarn BR, Batten A, et al. Interpreting treatment effects from clinical trials in the context of real-world risk information: end-stage renal disease prevention in older adults. JAMA Intern Med. 2014;174(3):391-7.
4. Uhlig K, Leff B, Kent D, et al. A framework for crafting clinical practice guidelines that are relevant to the care and management of people with multimorbidity. J Gen Intern Med. 2014;29(4):670-679.
5. Zulman DM, Sussman JB, Chen X, Cigolle CT, Blaum CS, Hayward RA. Examining the evidence: a systematic review of the inclusion and analysis of older adults in randomized controlled trials. J Gen Intern Med. 2011;26(7):783-790.
6. Fried TR, Tinetti ME, Iannone L, O'Leary JR, Towle V, Van Ness PH. Health outcome prioritization as a tool for decision making among older persons with multiple chronic conditions. Arch Intern Med. 2011;171(20):1854-1856.
7. Montori VM, Brito JP, Murad MH. The optimal practice of evidence-based medicine: incorporating patient preferences in practice guidelines. JAMA. 2013;310(23):2503-2504.

8. Bayliss EA, Bonds DE, Boyd CM, et al. Understanding the context of health for persons with multiple chronic conditions: moving from what is the matter to what matters. Ann Fam Med. 2014;12(3):260-269.

9. Tran VT, Harrington M, Montori VM, Barnes C, Wicks P, Ravaud P. Adaptation and validation of the Treatment Burden Questionnaire (TBQ) in English using an internet platform. BMC Med. 2014;12:109.
10. Boyd CM, Wolff JL, Giovannetti E, et al. Healthcare task difficulty among older adults with multimorbidity. Med Care. 2014;52 Suppl 3:S118-125.
11. Bynum JPW, Meara ER, Chang CH, Rhoads JM, Bronner KK, Our parents, ourselves: Health care for an aging population. Lebanon, NH: The Dartmouth Institute of Health Policy and Clinical Practice, 2016.
12. Jowsey T, Yen L, Mathews P. Time spent on health related activities associated with chronic illness: a scoping literature review. BMC Public Health. 2012;12(1):1044.
13. Soni A. Out-of-Pocket Expenditures for Adults with Health Care Expenses for Multiple Chronic Conditions, U.S. Civilian Noninstitutionalized Population. Statistical Brief (Medical Expenditure Panel Survey (US). Rockville (MD): Agency for Healthcare Research and Quality (US). 2017. STATISTICAL BRIEF #498.
14. Lorgunpai SJ, Grammas M, Lee DSH, McAvay G, Charpentier P, Tinetti ME. Potential therapeutic competition in community-living older adults in the U.S.: Use of medications that may adversely affect a coexisting condition. PLoS ONE. 2014. 9(2): e89447.
15. Institute of Medicine (IOM). Crossing the Quality Chasm: A New Health System for the 21st Century. Washington, D.C: National Academy Press; 2001.
16. Tinetti ME, Naik AD, Dodson JA. Moving from disease-centered to patient goals–directed care for patients with multiple chronic conditions: Patient value-based care. JAMA Cardiol 2016;1(1):9-10.
17. Tinetti ME, Esterson J, Ferris R, Posner P, Blaum CS. Patient priority directed decision-making and care for older adults with multiple chronic conditions. Clin Geriatr Med. 2016; 32(2):261-275.
18. Ferris R, Blaum C, Kiwak E, et al. Perspectives of patients, clinicians, and health system leaders on changes needed to improve the health care and outcomes of older adults with multiple chronic condition. J Aging Health. 2018;20(5):778-799.
19. Naik AD, Dindo L, Van Liew J, et al. Development of a clinically-feasible process for identifying patient health priorities. J Am Geriatr Soc. 2018; 66(10):1872-1879.
20. Blaum C, Rosen J, Naik AD, et al. Feasibility of Implementing patient priorities-aligned care for patients with multiple chronic conditions. J Am Geriatr Soc. 2018; 66(10):2009-2016.
21. Emily M. Mitchell. Concentration of Health Expenditures in the U.S. Civilian Noninstitutionalized Population, 2014. STATISTICAL BRIEF #497.
22. Griffith LE, Gruneir A, Fisher K, Panjwani D, Gafni A, Patterson C, Markle-Reid M, Ploeg J. Insights on multimorbidity and associated health service use and costs from three population-based studies of older adults in Ontario with diabetes, dementia and stroke. BMC Health Serv Res. 2019 May 16;19(1):313. doi: 10.1186/s12913-019-4149-3.

23. Coulter A, Entwistle VA, Eccles A, Ryan S, Shepperd S, Perera R. Personalized care planning for adults with chronic or long-term health conditions. Cochrane Database Syst Rev. 2015;(3):CD010523.
24. Smith SM, Wallace E, O'Dowd T, Fortin M. Interventions for improving outcomes in patients with multimorbidity in primary care and community settings. Cochrane Database Syst Rev. 2016; 3:CD006560.
25. Salisbury C, Man M-S, Bowers P, et al. Management of multimorbidity using a patient-centered care model: a pragmatic cluster-randomised trial of the 3D approach. Lancet. 2018; 392 (10141):41-50.
26. Naik AD, Martin LA, Moye J, Karel MJ. Health values and treatment goals of older, multimorbid adults facing life-threatening illness. J Am Geriatr Soc. 2016;64(3):625-631.
27. Childers JW, Back AL, Tulsky JA, Arnold RM. REMAP: A framework for goals of care conversations. J Oncol Pract. 2017;13(10): e844-e850.
28. Austin CA, Mohottige D, Sudore RL, Smith AK, Hanson LC. Tools to promote shared decision making in serious Illness: A systematic review. JAMA Intern Med. 2015;175(7):1213-1222.
29. Sudore RL, Knight SJ, McMahan RD, et al. A novel website to prepare diverse older adults for decision making and advance care planning: a pilot study. J Pain Symptom Manage. 2014;47(4):674-686.
30. Bernacki RE, Block SD. American College of Physicians High Value Care Task Force. Communication about serious illness care goals: a review and synthesis of best practices. JAMA Intern Med. 2014;174(12):1994-2003.
31. MacKenzie MA, Smith-Howell E, Bomba PA, Meghani SH. Respecting Choices and Related Models of Advance Care Planning: A Systematic Review of Published Evidence. Am J Hosp Palliat Med. 2018; 35(6):897–907.
32. Naik AD, Palmer N, Petersen NJ, et al. Comparative effectiveness of goal setting in diabetes mellitus group clinics: randomized clinical trial. Arch Intern Med. 2011;171(5):453-459.
33. Coulter A, Entwistle VA, Eccles A, Ryan S, Shepperd S, Perera R. Personalized care planning for adults with chronic or long-term health conditions. Cochrane Database Syst Rev. 2015;(3):CD010523.
34. Rockwood K, Stadnyk K, Carver D, et al. A clinimetric evaluation of specialized geriatric care for rural dwelling, frail older people. J Am Geriatr Soc. 2000;48(9):1080-1085.
35. Toto PE, Skidmore ER, Terhorst L, Rosen J, Weiner DK. Goal Attainment Scaling (GAS) in geriatric primary care: a feasibility study. Arch Gerontol Geriatr. 2015;60(1):16-21.
36. Tinetti ME, Naik AD, Dindo L, et al. Association of Patient Priorities–Aligned Decision-Making With Patient Outcomes and Ambulatory Health Care Burden Among Older Adults With Multiple

Chronic Conditions: A Nonrandomized Clinical Trial. JAMA Intern Med. 2019. doi:https://doi.org/10.1001/jamainternmed.2019.4235

37. Rosner, B. 2011. Fundamentals of Biostatistics, Seventh Edition. Brooks/Cole. Boston, MA.
38. Hsieh, F.Y., Block, D.A., and Larsen, M.D. 1998. 'A Simple Method of Sample Size Calculation for Linear and Logistic Regression', Statistics in Medicine, Volume 17, pages 1623-1634.
39. Hays RD, Bjorner JB, Revicki DA, Spritzer KL, Cella D. Development of physical and mental health summary scores from the patient-reported outcomes measurement information system (PROMIS) global items. Qual Life Res. 2009; 18(7): 873-880.
40. Brandt Jason, Spencer Miriam, Folstein Marshal F. The Telephone Interview for Cognitive Status. Neuropsychiatry, Neuropsychology, and Behavioral Neurology. 1988;1:111–17.
41. Forcino RC, Barr PJ, O'Malley AJ, et al. Using CollaboRATE, a brief patient- reported measure of shared decision making: Results from three clinical settings in the United States. Health Expect.2018;21(1):82–89.
42. Tran VT, Harrington M, Montori VM, Barnes C, Wicks P, Ravaud P. Adaptation and validation of the Treatment Burden Questionnaire (TBQ) in English using an internet platform. BMC Med. 2014;12:109.
43. Bynum JPW, Meara ER, Chang CH, Rhoads JM, Bronner KK, Our parents, ourselves: Health care for an aging population. Lebanon, NH: The Dartmouth Institute of Health Policy and Clinical Practice, 2016.
44. Tinetti ME, Naik AD, Dindo L, et al. Association of Patient Priorities–Aligned Decision-Making With Patient Outcomes and Ambulatory Health Care Burden Among Older Adults With Multiple Chronic Conditions: A Nonrandomized Clinical Trial. JAMA Intern Med. 2019. doi:https://doi.org/10.1001/jamainternmed.2019.4235

Appendix.0

Complete list of chronic conditions

Acquired Hypothyroidism
Acute Myocardial Infarction
Alzheimer's Disease
Alzheimer's Disease and Related Disorders or Senile Dementia
Anemia
Asthma
Atrial Fibrillation
Benign Prostatic Hyperplasia*
Cataract
Chronic Kidney Disease
Chronic Obstructive Pulmonary Disease and Bronchiectasis
Depression
Diabetes
Glaucoma
Heart Failure
Hip/Pelvic Fracture
Hyperlipidemia
Hypertension
Ischemic Heart Disease
Osteoporosis
RA/OA (Rheumatoid Arthritis/ Osteoarthritis)
Stroke / Transient Ischemic Attack**
Female / Male Breast Cancer
Colorectal Cancer
Prostate Cancer
Lung Cancer
Endometrial Cancer

Appendix 1. PPC components aligned with existing CCF roles

PPC Priorities Facilitator's key components	Care Coordinator's job description (excerpt from T99128 - Care Coordinator)		Patient Liaison Program Coordinator's job description (excerpt from D99797 - Patient Liaison Program Coordinator)
Patient identification	• Identifies which patients in the specialty care practice have ongoing care coordination needs for their specialty condition.<br><br>• Utilizes technological tools (registries, patient lists, care team tab, etc.) to manage populations.		• Proactively completes intentional rounding to meet new patients, promoting available programs and services.
Patient priority assessment	• Utilizes assessment skills and risk assessment tools to identify patients with actual or potential care needs that would require care coordination.<br><br>• Conducts comprehensive clinical assessments that include disease-specific, age-specific, medical, behavioral pharmacy, social and end of life needs of each patient.<br><br>• Ensures care gaps are closed around specialty disease/chronic disease/surgical episodes.		• Facilitates communication or responses to patient needs; ensures positive working relationship between physicians, employees, patients and families; supports highest quality of patient standards.<br><br>• Identifies, evaluates, and resolves patient complaints.<br><br>• Removes barriers to care, and facilitates coordination of care.

Collaboration with other specialty providers	• Works collaboratively with interdisciplinary team to develop goals and plan interventions to maximize patient outcomes.<br>• Partners with other care coordinator teams such as primary and transitional care social work, rehabilitation, pharmacy, palliative care and others		• Works with other areas within the Institute and across the enterprise to implement services that meet the needs of the patient or the patient's family.
On-going patient support	• Monitors patient compliance with plan of care.<br>• Performs reassessments regarding patient progress toward goals and updates plan of care as appropriate.<br>• Serves as primary patient contact for team related to condition/surgical episode and facilitates access to services.<br>• Coordinates members of the patient care team.<br>• Works with the patient and family to assess current knowledge, health literacy, and readiness to change, utilizing teach back to assess level of knowledge.		• Provides education as needed.<br>• Captures patterns of successes and challenges within the patient experience and enter into a data base to perform analysis that will drive improvements to patient services.<br>• Involved in the implementation of new processes that will guide patients to the appropriate resources and support services they need to improve the patient experience.
