# Supplementary material for: Patient Priorities–Aligned Care for Older Adults With Multiple Conditions: A Nonrandomized Controlled Trial
Source: JAMA Netw Open. 2024 Jan 23;7(1):e2352666. doi: 10.1001/jamanetworkopen.2023.52666 (PMC10807252; doi:10.1001/jamanetworkopen.2023.52666)
Supplement: Supplement 2. — eAppendix. Supplemental Methods eReferences [file jamanetwopen-e2352666-s002.pdf]

Supplemental Online Content

Tinetti ME, Hashmi A, Ng H, et al. Patient priorities–aligned care for older adults with multiple conditions: a nonrandomized controlled trial. *JAMA Netw Open*. 2024;7(1):e2352666. doi:10.1001/jamanetworkopen.2023.52666

eAppendix. Supplemental Methods

eReferences

This supplemental material has been provided by the authors to give readers additional information about their work.

© 2024 Tinetti ME et al. *JAMA Network Open.*

eAppendix. Supplemental Methods

Study design and setting

The study followed a nonrandomized controlled trial design.[1] One primary care site within Cleveland Clinic's multisite Primary Care Practice was selected as the PPC site. A matching procedure was used to identify the UC site to balance site-level variables. The matching procedure calculates the Mahalanobis distance metric with site-level matching variables and accounts for the variances and correlations of the variables across sites.[2,3] Matching variables included percent of patients: 1) ≥65 years; 2) ≥65 years and Nonwhite; 3) ≥65 years and dual Medicare-Medicaid recipients. Among the 11 potential UC sites, the final UC site was identified as the optimal match to the PPC site in term of the specified distance metric. The manuscript follows the TREND reporting guide for nonrandomized trials.

Participants and enrollment

Clinicians. Six of the seven primary care providers (PCPs) providing care at the PPC site participated, including four physicians and two advanced practice providers (APPs). A PPC site physician (HN), served as clinical champion, supporting his colleagues in implementing PPC. One PCP, who was retiring, declined. All nine PCPs (six physicians and three APPs) at the UC site participated.

Patients. Potentially eligible patients were those cared for by the participating PCPs. Inclusion criteria included age ≥65 plus any of ≥3 chronic conditions; ≥10 medications; ≥2 specialist visits, >2 ED visits or >1 hospitalization or ≥10 hospital days, or received care coordination services, in the past year. Exclusion criteria included non-English speaking**,** meeting hospice criteria; advanced dementia or moderate to profound intellectual disabilities; or long-term nursing home resident. Administrative data were used to identify patients of the participating PCPs meeting these criteria who had a scheduled visit within six weeks. Potential participants were invited through the electronic health record (EHR) if they had an active patient portal or by letter if not. They then were contacted by phone to explain the project, determine final eligibility (primarily cognition, very advanced illness, language, nursing home residence), and obtain consent. Eligible and consenting participants cared for by UC PCPs completed the baseline interview (described below) during this call as did PPC participants initially. The protocol was modified so that health priorities identification occurred during the first call; the baseline interview could occur that day or be scheduled for another day for PPC participants. The enrollment date was the date of the baseline interview except for PPC participants who declined the baseline interview but completed priorities identification; enrollment began with priorities identification for these latter participants. Enrollment occurred between August 21, 2020 – May 14, 2021. Follow ups continued through February 26, 2022. The flow diagram of PPC and UC participants is displayed in the **Figure**. The Cleveland Clinic Institutional Review Board approved the study. Oral consent was obtained from study participants.

Patient Priorities Care Intervention

The development of PPC has been described previously.[4-7] We used a Practice Change Framework (e.g., leadership support, clinical champions, training, workflow support, health information technology (HIT) enhancements, collaborative learning) and Plan-Do-Study-Act (PDSA) cycles to implement PPC.[8-10] Patient Priorities Care begins with guidance from a facilitator who helps patients construct their health priorities. Facilitators can be drawn from a range of health professions, such as social work, nursing, psychology, and medicine with motivational interviewing or related therapeutic skills. The clinician-facilitators who participated in the current study included four members of the healthcare team (an APP, nurse navigator, care coordinator, and geriatrician) who completed previously described training.[4,7] Facilitators prepared for training by reviewing the patient and facilitator manuals. Training began with a virtual session that included instructions on use of the manuals as guides for the patient priorities identification, followed by role playing with feedback provided by the PPC investigator team. Facilitators received ongoing feedback through four teleconferences with the PPC investigator team. The aim of training was to ensure that the process results in specific, actionable and realistic, goals and specific care preferences that can inform decision making and prepare patients to engage with their clinicians around health priorities aligned care. During phone visits, the facilitators guided patients, and caregivers when desired, through identification of: 1) core values; 2) most desired specific, actionable and realistic outcome goals; 3) healthcare preferences (i.e. aspects of current care (medications, healthcare visits, tests, procedures, self-management tasks, supportive services) that they felt were doable and helpful and those aspects they felt didn't help or were bothersome; and 4) the top priority (i.e., the symptom, health problem, or healthcare activity) they most wanted their clinicians to focus on because it was so bothersome or impeding achievement of their most desired outcome

© 2024 Tinetti ME et al. *JAMA Network Open.*

goal).[4,7,11]The completed Patient Health Priorities template, including this information, was placed in easily accessible locations in the EHR.

Participating PCPs at the PPC site completed a two-hour virtual training, led by the PPC investigator team (MT, JE, AN, LD), based on previously described PPC training.[7] The session provided the rationale for patient priorities aligned care for older adults with MCCs, presented the decisional strategies listed below, introduced examples of translating patients health priorities into care decisions, discussed incorporating PPC into the clinic workflow, and addressed questions and concerns raised by the PCPs. The PCPs and health priorities facilitators then piloted PPC for two months before enrollment began. The PCPs participated in eight 15–30-minute, case-based huddles over twelve month, facilitated by the Cleveland Clinic PPC site principal investigator (AH) and clinical champion (HN) and attended by members of the PPC team (MT, JE, AN, LD).[12] These huddles enabled everyone to share their challenges, experiences and suggestions concerning patient-priorities aligned decision-making. PCPs were alerted to the presence of their patient's health priorities template in the EHR and discussed them during the initial visit after priorities identification. For this and subsequent visits, PCPs were instructed to use patient priorities decisional strategies (i.e., use patients' health priorities as focus of communication and decision-making; use serial trials to start, stop or continue care based on achieving health outcome goals and consistent with healthcare preferences; align decision-making among clinicians when there are different perspectives or recommendations) to decide with patients what care to stop, start or continue.[6,13] PPC decisional guidance also included strategies for trouble shooting common challenges such as when patients' health outcome goals were not achievable either because of health status or because patients were not able or willing to receive the healthcare necessary to achieve their outcome goals.[14]

Outcomes

The primary patient reported outcomes (PROs) included: 1) perceived treatment burden measured by the Treatment Burden Questionnaire (TBQ) (score range 0-150, Cronbach's alpha=0.90);[15] 2) achievement of desired activities measured by PROMIS Ability to Participate in Social Roles and Activities Short Form 6a (score range 6-30; Cronbach's alpha = 0.98);[16] and 3) CollaboRATE (Cronbach's alpha=0.89; score 0-100; dichotomized 100 vs. <100 with 100 being the percent of participants who reported the top score of all three items)[17] The secondary outcome was the Cleveland Clinic Accountable Care Organization (ACO) shared prescribing decision-making quality measure, *"When starting a new medication, did your provider ask what you thought was best for you"*. The PROs were ascertained by phone at baseline and after nine months follow-up; PROMIS Social Roles and Activities was ascertained only at the nine-month follow-up.

Nonhealthy Days, a primary outcome, was a modified inverse of the Health Days at Home measure, similar to Healthcare Contact Days.[18-21] Nonhealthy Days were the number of days in which persons were dead or in the hospital, emergency department (ED), nursing home (NH) or undergoing ambulatory procedures requiring several hours for completion and recovery (e.g., endoscopy, ambulatory surgeries). Persons were included in only one category per day (death>hospital>ED>procedure>NH). These data were ascertained from EHR covering 90 days prior to, and 365 days post, enrollment.

Outcomes were ascertained by assessors blinded to site; data entry forms did not include PCP site.

Covariates

Sociodemographic data, 18 chronic conditions (based on Centers for Medicare and Medicaid Services' Chronic Conditions Warehouse ICD-10 algorithms), and medications were ascertained from the EHR.[22] During the baseline interview, memory was assessed with 5-item recall.[23] The physical and mental health measures from the patient-reported outcomes measurement information system (PROMIS) global, were included in the baseline and follow-up interviews.[24] Baseline characteristics included in the propensity score included age, sex, race, living situation, education, health insurance, number of chronic conditions and medications, presence of heart failure or chronic lung disease, 5-item recall, baseline physical and mental health, treatment burden, CollaboRATE, and ACO shared prescribing decision-making quality measure, and Nonhealthy Days in the three months prior to enrollment.

Analysis

In our original proposal, we assessed the power to detect clinically relevant differences in PROs and categories of healthcare utilization. We calculated minimal detectable differences for the primary PPC outcomes based on a sample size of 500 participants (250 PPC and 250 UC) that assumed 80% power, a two-tailed alpha of 0.05, and an R-squared of 0.2 from other covariates. Assuming a standard deviation of 24.1 at pre-test and 17.6 at post-test and a correlation between measurement pairs of 0.55 (based on pilot data) allows us to detect a between-group difference in mean change in the TBQ score from pre-test to post-test of -4.6 points. We did not recalculate power for the reduced sample size (**See effects of COVID on execution of the study**).

© 2024 Tinetti ME et al. *JAMA Network Open.*

Missing data were handled by using multiple imputation using the fully conditional specification procedure in SAS.[25] Those who died during the study prior to follow-up were assigned the worst score for the outcome prior to imputation.

We estimated propensity scores using logistic regression for each imputed dataset with the PSMATCH procedure (SAS, version 9.4; SAS Institute INC). Since the matching procedure only ensures balance in the site-level characteristics, we used inverse probability weighting based on propensity scores to ensure balance in measured patient-level characteristics across arms[26-29] Balance was evaluated by comparing the weighted distribution of the covariates using absolute standardized mean differences of .25 or less.[29]

To ensure objectivity, the same multiple imputed and propensity weighted datasets were used for all analyses and all outcomes. The set of patient-level variables included in the propensity score model were chosen by the study team as the minimum sufficient set of potential confounders that differ between sites and potentially correlate with the outcomes of interest. Each patient in the PPC site was weighted by the inverse of the estimated propensity score to receive PPC, and each patient in the UC site was weighted by the inverse of one minus the estimated propensity score. A subset of these potential confounders were further adjusted for in the outcome regression model to address residual imbalance that may persist after weighting. For the imputation model, we included all variables used in the propensity score analyses as well as all outcomes and the treatment arm.

For the raw data we compared the distribution of key covariates across treatment condition in the raw data using chi-square or t-tests as appropriate. We also assessed the association of arm and key covariates in the weighted and imputed data in a model with covariate as the dependent variable and arm as predictor, within a linear or logistic regression model as appropriate. Propensity score weighted multivariable linear regression models were used to examine the strength and significance of the association between PCP site and 1) PROs at baseline and follow-up; and 2) number of Nonhealthy Days within 365 days of baseline interview and 30 days prior to baseline interview. The dichotomized CollaboRATE scale and ACO shared prescribing decision-making quality measure were analyzed using propensity score weighted logistic regression. All outcome models were adjusted for the corresponding baseline value (90-day pre-enrollment for Nonhealthy Days) as appropriate, demographic and clinical characteristics, and the estimated propensity weights.[30] Rather than a complete case analysis, we combined multiple imputation with propensity score weighting for our primary analyses, and addressed potential confounding and selection bias to the extent possible. Any residual imbalance after propensity score weighting was further adjusted for by multivariable regression to enhance the robustness of findings.[31,32] Robust sandwich variance was used for analysis of each complete data, and Rubin’s formula was used to combine estimates from multiply imputed full datasets into a single set of results using the MIANALYZE procedure in SAS.[25]

Effects of COVID-19 on execution of the study

Protocol challenges resulting from the COVID-19 pandemic included: 1) moving training from in-person to virtual; 2) several month delay in start of enrollment and anticipated deployments of PCPs and team members to COVID-related duties resulted in a change in targeted enrollment from 500 to 250; 3) actual COVID-related deployments led to missing potentially eligible participants (see **Figure**); 4) patients received multiple check in phone calls from Cleveland Clinic teams; some patients expressed difficulty distinguishing the health priorities call from other calls; 5) facilitators and baseline interviewers were working at home and calling from their private lines, resulting in fewer calls answered due to unfamiliar numbers on the caller ID; 6) PCP appointments were rescheduled during COVID surges, leading to temporal gaps between priorities identification and PCP visits; 7) deferred care for chronic conditions.

© 2024 Tinetti ME et al. *JAMA Network Open.*

eReferences

1. Handley MA, Lyles CR, McCulloch C, Cattamanchi A. Selecting and improving quasi-experimental designs in effectiveness and implementation research. Annu Rev Public Health. 2018; 39:5-25.
2. Rubin DB. Using multivariate matched sampling and regression adjustment to control bias in observational studies. J Am Statist Assoc.1979; 74:318–328.
3. Zhao Z. Using matching to estimate treatment effects: Data requirements, matching metrics, and monte carlo evidence. Rev Econom and Statist. 2004; 86(1):91-107.
4. Naik AD, Dindo L, Van Liew J, et al. Development of a clinically feasible process for identifying patient health priorities. J Am Geriatr Soc. 2018; 66(10):1872-1879.
5. Blaum C, Rosen J, Naik AD, et al. Feasibility of implementing patient priorities-aligned care for patients with multiple chronic conditions. J Am Geriatr Soc. 2018; 66(10):2009-2016.
6. Tinetti ME, Dindo L, Smith CD, et al. Challenges and strategies in patients' health priorities-aligned decision-making for older adults with multiple chronic conditions. [Published online June 10, 1019]. PLoS ONE. 2019; https://doi.org/10.1371/journal.pone.0218249.
7. Tinetti ME, Naik AD, Dindo L, et al. Association of patient priorities-aligned decision-making with patient outcomes and ambulatory health care burden among older adults with multiple chronic conditions. JAMA Int Med. 2019. 7;179(12):1688-1697.
8. Lau R, Stevenson F, Ong BN et al. Achieving change in primary care—causes of the evidence to practice gap: systematic reviews of reviews. Implement Sci. 2016; 11(1):40.
9. Noël PH, Lanham HJ, Palmer RF et al. The importance of relational coordination and reciprocal learning for chronic illness care within primary care teams. Health Care Manage Rev. 2013; 38(1):20-28.
10. Batalden PB, Stoltz PK. A framework for the continual improvement of health care: building and applying professional and improvement knowledge to test changes in daily work. J Comm J Qual Improv. 1993; 19(10):424-447.
11. Davenport C, Ouellet J, Tinetti ME. Use of the patient-identified top priority in care decision-making for older adults with multiple chronic conditions. [Published online October 1, 2021]. JAMANetwOpen. 2021. 4(10):e2131496. doi: 10.1001/jamanetworkopen.2021.31496.
12. Ouellet JA, Kiwak E, Tinetti ME, et al. A qualitative study of coaching patient priorities aligned decision making through virtual case-based huddles. J Am Geriatr Soc. 2023; DOI: 10.1111/jgs.18609.
13. Patient Priorities Care Decisional Guidance at http://decisionguide.patientprioritiescare.org. Accessed August 14, 2023.
14. Troubleshooting: Common challenges in aligning decisions with patients' health priorities. At https://patientprioritiescare.org/decisionguide/troubleshooting. Accessed August 14, 2023.
15. Tran VT, Harrington M, Montori VM, Barnes C, Wicks P, Ravaud P. Adaptation and validation of the Treatment Burden Questionnaire (TBQ) in English using an internet platform. BMC Med. 2014; December. 12:109.
16. Hahn EA, Kallen MA, Jensen RE, et al. Measuring social function in diverse cancer populations: Evaluation of measurement equivalence of the Patient Reported Outcomes Measurement Information System® (PROMIS®) Ability to Participate in Social Roles and Activities short form. Psychol Test Assess Model. 2016; 58(2):403–421.
17. Barr PJ, Forcino RC, Thompson R, et al. Evaluating CollaboRATE in a clinical setting: analysis of mode effects on scores, response rates and costs of data collection. Published online March 1, 2017. BMJ open. 2017;7(3):e014681.
18. Burke LG, Orav EJ, Zheng J, Jha AK. Healthy Days at home: A novel population-based outcome measure. [published online November 8, 2019]. Healthc (Amst). 2020; 8(1):100378. doi: 10.1016/j.hjdsi.2019.100378.
19. Groff C, Colla CH, Lee TH. Days spent at home - a patient-centered goal and outcome. N Engl J Med. 2016; 375 (17):1610-161.
20. Lee H, Shi SM, Kim DH. Home time as a patient-centered outcome in administrative claims data. J Am Geriatr Soc. 2019; 67(2):347-351.
21. Bynum JPW, Meara ER, Chang CH, Rhoads JM, Bronner KK. Our parents, ourselves: Health care for an aging population. Lebanon, NH: The Dartmouth Institute of Health Policy & Clinical Practice, 2016.
22. Chronic Conditions Warehouse. Accessed at ccw-chronic-condition-algorithms%20(1).pdf.
23. Nasreddine ZS, Phillips NA, Bédirian V, et al. The Montreal Cognitive Assessment, MoCA: A brief screening tool for mild cognitive impairment. J Am Geriatr Soc. 2005. 53(4): 695-699.

© 2024 Tinetti ME et al. *JAMA Network Open.*

24. Hays RD, Bjorner JB, Revicki DA, Spritzer KL, Cella D. Development of physical and mental health summary scores from the patient-reported outcomes measurement information system (PROMIS) global items. Qual Life Res. 2009; 18(7):873-880.
25. Rubin DB. Multiple imputation for nonresponse in surveys. In Book Series: Wiley Series in Probability and Statistics. [Published online June 9, 1987[. ISBN:9780470316696 DOI:10.1002/9780470316696.
26. Stuart EA. Matching methods for causal inference: A review and a look forward. Stat. Sci. 2010; 25(1):1–21.
27. Austin PC. An introduction to propensity score methods for reducing the effects of confounding in observational studies. Multivariate Behav Res. 2011; 46(3):399–424.
28. Austin PC, Stuart EA. Moving towards best practice when using inverse probability of treatment weighting (IPTW) using the propensity score to estimate causal treatment effects in observational studies. Stat. Med. 2015; 34(28):3661–3679.
29. Rubin DB. Using propensity scores to help design observational studies: Application to the tobacco litigation," Matched Sampl. Causal Eff. 2006; 365–382. doi: 10.1017/CBO9780511810725.030.
30. Kang JDY, Schafer JL. Demystifying double robustness: A comparison of alternative strategies for estimating a population mean from incomplete data. Stat Sci. 2007; 22:523-539.
31. Leyrat C, Seaman SR, White IR, et al. Propensity score analysis with partially observed covariates: how should multiple imputation be used?. Statistical Methods in Medical Research. 2019. 28(1):3-19.
32. Funk MJ, Westreich D, Wiesen C, Stürmer T, Brookhart MA, Davidian M. (2011). Doubly robust estimation of causal effects. American Journal Epidemiol. 2011. 173(7): 761-767.

© 2024 Tinetti ME et al. *JAMA Network Open.*
