# Supplementary material for: Patient Priorities–Aligned Care for Older Adults With Multiple Conditions: A Nonrandomized Controlled Trial
Source: JAMA Netw Open. 2024 Jan 23;7(1):e2352666. doi: 10.1001/jamanetworkopen.2023.52666 (PMC10807252; doi:10.1001/jamanetworkopen.2023.52666)
Supplement: Supplement 3. — Data Sharing Statement [file jamanetwopen-e2352666-s003.pdf]

Data Sharing Statement

Tinetti. Patient Priorities–Aligned Care for Older Adults With Multiple Conditions. *JAMA Netw Open*. Published January 23, 2024. doi:10.1001/jamanetworkopen.2023.52666

Data
Data available: Yes
Data types: Deidentified participant data, Data dictionary
How to access data: Margaret.doyle@yale.edu
When available: With publication

Supporting Documents
Document types: Statistical/analytic code
How to access documents: Margaret.doyle@yale.edu
When available: With publication

Additional Information
Who can access the data: Anyone with appropriate reason for request
Types of analyses: any purpose
Mechanisms of data availability: signed agreement
